# Integration of *Lupinus angustifolius* L. (narrow-leafed lupin) genome maps and comparative mapping within legumes

**DOI:** 10.1007/s10577-016-9526-8

**Published:** 2016-05-11

**Authors:** Katarzyna Wyrwa, Michał Książkiewicz, Anna Szczepaniak, Karolina Susek, Jan Podkowiński, Barbara Naganowska

**Affiliations:** 1 Institute of Plant Genetics of the Polish Academy of Sciences, Strzeszyńska 34, Poznań, 60-479 Poland; 2 Institute of Bioorganic Chemistry of the Polish Academy of Sciences, Z. Noskowskiego 12/14, Poznań, 61-704 Poland

**Keywords:** *Lupinus angustifolius*, chromosome markers, integrated map, rDNA, centromeres, synteny

## Abstract

Narrow-leafed lupin (*Lupinus angustifolius* L.) has recently been considered a reference genome for the *Lupinus* genus. In the present work, genetic and cytogenetic maps of *L. angustifolius* were supplemented with 30 new molecular markers representing lupin genome regions, harboring genes involved in nitrogen fixation during the symbiotic interaction of legumes and soil bacteria (Rhizobiaceae). Our studies resulted in the precise localization of bacterial artificial chromosomes (BACs) carrying sequence variants for early nodulin 40, nodulin 26, nodulin 45, aspartate aminotransferase P2, asparagine synthetase, cytosolic glutamine synthetase, and phosphoenolpyruvate carboxylase. Together with previously mapped chromosomes, the integrated *L. angustifolius* map encompasses 73 chromosome markers, including 5S ribosomal DNA (rDNA) and 45S rDNA, and anchors 20 *L. angustifolius* linkage groups to corresponding chromosomes. Chromosomal identification using BAC fluorescence in situ hybridization identified two BAC clones as narrow-leafed lupin centromere-specific markers, which served as templates for preliminary studies of centromere composition within the genus. Bioinformatic analysis of these two BACs revealed that centromeric/pericentromeric regions of narrow-leafed lupin chromosomes consisted of simple sequence repeats ordered into tandem repeats containing the trinucleotide and pentanucleotide simple sequence repeats AGG and GATAC, structured into long arrays. Moreover, cross-genus microsynteny analysis revealed syntenic patterns of 31 single-locus BAC clones among several legume species. The gene and chromosome level findings provide evidence of ancient duplication events that must have occurred very early in the divergence of papilionoid lineages. This work provides a strong foundation for future comparative mapping among legumes and may facilitate understanding of mechanisms involved in shaping legume chromosomes.

**Electronic supplementary material:**

The online version of this article (doi:10.1007/s10577-016-9526-8) contains supplementary material, which is available to authorized users.

## Introduction

Plant nuclear genomes are organized into chromosomes that facilitate genome investigation (Heslop-Harrison and Schwarzacher 2011). Despite the increasing amounts of genetic, genomic, and transcriptomic data that are now available, little is known about plant chromosomal organization and evolution. Studies of entire sets of chromosomes (karyotype) using cytogenetic approaches have been important in identifying chromosome number, size, and morphology. In addition, cytogenetics is a useful tool for the distinction or characterization of species and inference of their evolution and relationships (Kato et al. 2005; Iovene et al. 2008; Figueroa and Bass 2010). Karyotype analyses, especially in species with symmetric karyotype and many chromosomes, allow the genome structure to be tracked (Paterson et al. 2000; Young et al. 2012; Dolezel et al. 2014). In species with well-established genomic resources for chromosome markers, the availability of whole-genome sequences has contributed significantly to the construction of chromosomal maps and the further assignment of the markers to physical/linkage groups (Sato et al. 2007). However, in species with numerous small and uniform chromosomes, genomic studies are still a challenge, especially when a completely assembled reference genome sequence is not yet available.

Fluorescence in situ hybridization (FISH), especially with large-insert bacterial artificial chromosomes (BACs) as molecular probes (BAC-FISH), has contributed greatly to plant genome analyses at the chromosomal level. FISH techniques have been widely used to generate landmarks for plant mitotic (Hasterok et al. 2006; Lamb et al. 2007; Hřibová et al. 2008) and meiotic (Lysak et al. 2001; Idziak et al. 2011) chromosomes. These cytogenetic markers are essential not only to distinguish particular chromosomes but also for assignment of genetic and chromosomal maps (Ellis and Poyser 2002; Ohmido et al. 2010) and alignment of genome sequence assemblies to chromosomes (Sun et al. 2013; Shearer et al. 2014). BAC-FISH has been applied for chromosome characterization and integrative mapping in many species including *Brassica oleracea*, *Phaseolus lunatus*, *Phaseolus vulgaris*, *Sorghum bicolor*, *Oryza sativa*, *Arabidopsis thaliana*, and *Solanum esculentum* (Jiang et al. 1995; Islam-Faridi et al. 2002; Kim et al. 2002; Koornneef et al. 2003; Howell et al. 2005; Koo et al. 2008; Bonifacio et al. 2012). The integration of *B. oleracea* cytogenetic and genetic linkage maps was achieved with 22 BAC probes representing 19 loci (Howell et al. 2002). FISH also allowed the simultaneous identification of all 10 sorghum chromosomes using 17 BAC probes (Kim et al. 2002). Similar approaches have been applied for karyotype analyses of legumes, such as common bean (Pedrosa-Harand et al. 2009; Fonseca et al. 2010) and soybean (Findley et al. 2010). Genetically anchored BAC clones were used both to identify individual chromosomes in metaphase spreads and to establish a complete FISH-based karyotyping cocktail that permitted simultaneous identification of all 20 soybean chromosome pairs (Findley et al. 2010). Genome maps that associate all the chromosomes to corresponding linkage groups have been constructed for a number of plant species (Feng et al. 2013) including *Medicago truncatula* (Schnabel et al. 2003), *Lotus japonicus* (Ohmido et al. 2010), and *P. vulgaris* (Fonseca et al. 2010).

An important step in karyotype analyses is determining the position and composition of the centromere region. Although the prominence of the role of centromeres during cell divisions is known, they are often overlooked during whole-genome studies at both the sequence and chromosomal levels. Centromere sequence organization studies have been performed to complement the understanding of the structure of higher plant genomes as well as to decipher their evolution dynamics (Neumann et al. 2015). Analyses of sequence data revealed that plant centromeres are rich in various types of repetitive DNA (He et al. 2015). Despite the conserved function of centromeres in all eukaryotic cells, large size variations and sequence divergence have been reported, even between closely related species (Melters et al. 2013; Neumann et al. 2015). Flowering plant centromeres in an individual genome are generally composed of the same types of DNA components, mainly large arrays of tandem satellite repeats and retrotransposons. A common characteristic of centromeric satellite repeats is their rapid divergence (Ma et al. 2007a; Melters et al. 2013), and polymorphisms have been found between individual chromosomes in the same genome, involving nucleotide sequence and copy numbers of repeats, as well as their spacing, order, and orientation (Yi et al. 2013). Whole-genome sequence annotations have revealed that the centromere composition in legumes also varies from one species to another (Cannon et al. 2009).

The legume family (Fabaceae) comprises approximately 19,500 species distributed in diverse ecological and geographical habitats (Lewis 2005). Extensive studies of a hallmark trait of 88 % of legume species, namely, their ability to develop root nodules and to fix atmospheric nitrogen in symbiosis with compatible Rhizobia, have been performed to better understand this process and for future improvement of legume crops. Genes involved in signal exchange between symbionts have been identified, primarily through genetic analysis and analytical chemistry. Transcriptomic analysis of complementary DNA (cDNA) libraries of *Glycine max* and *M. truncatula* suggested that 6555 genes may be involved in Fabaceae-Rhizobiaceae symbiosis (Brechenmacher et al. 2008). Among them, many transcription factors (Fedorova et al. 2002) and genes encoding early nodulin 40 (*ENOD40*) (Compaan et al. 2001), nodulin 26 (*NOD26*) (Wallace et al. 2006), nodulin 45 (*NOD45*) (Macknight et al. 1995), aspartate aminotransferase (*AAT*) (Griffith and Vance 1989), asparagine synthetase (*AS*) (Gaufichon et al. 2010), glutamine synthetase (*GS*) (Datta et al. 1991), and phosphoenolpyruvate carboxylase (*PEPC*) (Schuller et al. 1990) were predicted to play crucial roles during nitrogen fixation. The genus *Lupinus*, as a representative of legume species, exhibits many distinctive Fabaceae features, including the ability to fix atmospheric nitrogen by symbiosis with soil nitrogen-fixing bacteria (Graham and Vance 2003). *Lupinus angustifolius* (narrow-leafed lupin) is the most studied species within the genus, and it now became a reference organism in legume studies (Książkiewicz et al. 2015). The genetic toolbox of *L. angustifolius* encompasses molecular markers, transcriptome data, and a draft genome sequence, and the generation of thousands of molecular markers has facilitated the construction of genetic maps (Boersma et al. 2005; Kasprzak et al. 2006; Nelson et al. 2010; Gao et al. 2011; Kroc et al. 2014; Foley et al. 2015; Yang et al. 2015; Kamphuis et al. 2015). Cytogenetic investigation of the *L. angustifolius* genome has been hampered by the high number of small and similar chromosomes (2n = 40); nevertheless, some progress has been made. Analysis of the distribution of 5S and 45S ribosomal DNA (rDNA) loci allowed the identification of two pairs of chromosome markers for *L. angustifolius* (Naganowska and Zielinska 2002). A breakthrough in chromosomal studies in lupin was achieved using clones from a BAC library of the *L. angustifolius* nuclear genome for BAC-FISH (Leśniewska et al. 2011). Moreover, FISH-based mapping combined with genetic marker development resulted in the assignment of the first three linkage groups to corresponding chromosomes of *L. angustifolius* (Leśniewska et al. 2011), and BAC-FISH has been used to associate additional 10 linkage groups with chromosomes (Książkiewicz et al. 2013, 2015; Przysiecka et al. 2015). We also performed a complex *L. angustifolius* genome survey by combining the genetic and cytogenetic characterization of BAC clones, followed by sequence annotation and comparative studies of nine legume plants, which justified the conclusion that the genome regions studied had undergone whole-genome duplication (Książkiewicz et al. 2015).

Here, we describe the complete assignment of narrow-leafed lupin chromosomes to linkage groups on the most recent genetic map (Kamphuis et al. 2015). We identified, sequenced, and annotated two narrow-leafed lupin BAC clones as centromere-specific markers that may serve as a unique template for further studies of centromere composition within the *Lupinus* genus. We also present the comprehensive characterization of *L. angustifolius* genome regions that correspond to nine single-locus BAC clones and evidence their cross-genus microsynteny.

## Materials and methods

### Plant material

Seeds of *L. angustifolius* cv. Sonet (2n = 40), *L. albus* cv. Boros (2n = 50), and *L. luteus* cv. Mister (2n = 52) were obtained from the Polish Lupin GenBank in the Breeding Station Wiatrowo (Poznan Plant Breeders Ltd., Poland). Seeds of *L. angustifolius* mapping population were provided by Dr. Hua’an Yang (Department of Agriculture and Food Western Australia). Seeds of *Triticum aestivum* and *Nicotiana tabacum* were kindly provided by Dr. Michał Kwiatek (Department of Genomics, Institute of Plant Genetics PAS) and Dr. Tomasz Pniewski (Department of Biotechnology, IPG PAS), respectively.

### Screening of BAC library

A set of BAC clones was selected by screening the *L. angustifolius* cv. Sonet nuclear genome BAC library (Kasprzak et al. 2006) with sequence-specific probes, targeting *ENOD40*, *NOD26*, *NOD45*, and aspartate aminotransferase P2 (*AAT*-*P2*) genes. Target genes were chosen based on their involvement in pathway leading to symbiotic interaction of legume plants with nitrogen-fixing bacteria. Degenerated primers were anchored in gene sequences and designed using the following templates: *L. luteus ENOD40* (accession number AF352375), *G. max NOD26* (L12257), *L. angustifolius NOD45* (L12388), and *L. angustifolius AAT*-*P2* (L29258) (Online resource 1). Probes were generated using PCR with *L. angustifolius* genomic DNA as a template (25 ng DNA), Taq polymerase (Novazym, Poznan, Poland) supplied with 1× PCR buffer and 2.5 mM Mg^2+^, 0.16 mM dNTP, 0.25 μM of each primer, and deionized water up to 20 μL. The PCR amplification involved initial denaturation (94 °C, 5 min); then 40 cycles consisting of three steps, denaturation (94 °C, 30 s), annealing (54–58 °C, 40 s) (Online resource 1), elongation (72 °C, 55 s); and followed by final elongation (72 °C, 5 min). PCR products were purified with QIAquick PCR Purification Kit (Qiagen, Hilden, Germany) and sequenced. Probes were radioactively labeled by random priming with HexaLabel DNA Labeling Kit (Fermentas Waltham, MA, USA) with 50 μCi [α-32P]-dCTP. The hybridization of probes with the nuclear genome BAC library and verification of positive signals were carried out as previously described by Książkiewicz et al. (2013). Moreover, in order to integrate genetic and cytogenetic maps of all narrow-leafed lupin chromosomes, the research material was supplemented with 13 clones selected from other project concerning analysis of cytosolic glutamine synthetase (*GS1*), *PEPC*, and *AS* genes (Wyrwa, unpublished), genes encoding enzymes indispensable in active symbiosome during nitrogen fixation process.

### BAC-end and BAC insert sequencing

Selected BAC clones were isolated with PhasePrep BAC DNA Kit (Sigma Aldrich, St. Louis, USA) and subjected to BAC-end sequencing on the ABI PRISM 3130 XL Genetic Analyzer (Applied Biosystems, Foster City, USA) using the following pIndigoBAC5 sequencing primers:

- 3′-GGATGTGCTGCAAGGCGATTAAGTTGG,
- 5′-CTCGTATGTTGTGTGGAATTGTGAGC.

BAC-end sequences (BESs) were analyzed and manually edited with Chromas Lite software (Technylesium, South Brisbane, Australia).

The whole-insert sequencing was performed by Miseq platform (Illumina, San Diego, USA) in paired-end 2 × 250-bp approach (Genomed, Warsaw, Poland). BAC clone sequence assembly was performed with the use of CLCGenomicWorkbench (v7.0.4) software under default parameters (automatic word size, yes; automatic bubble size, yes; minimum contig length, 500; auto-detect paired distances, yes; perform scaffolding, yes; map reads back to contigs, yes; mismatch cost, 2; insertion cost, 3; deletion cost, 3; length fraction, 0.5; similarity fraction, 0.8; update contigs, yes).

### Functional annotation of BAC-end and whole-BAC sequences

Obtained sequence data were functionally annotated using nucleotide and protein BLAST algorithms (NCBI, http://www.ncbi.nlm.nih.gov/) in order to identify genes (databases DNA and protein sequences from the EMBL Nucleotide Sequence Database, GenBank, DNA Database of Japan, RCSB Protein Data Bank, Swiss-Prot, Protein Information Resource, and Protein Research Foundation) as well as repetitive sequences (databases RepBase, TIGR, and MIPS Plant Repeats Collections) (Ouyang and Buell 2004; Spannagl et al. 2007). The BLAST algorithm was optimized for highly similar sequences (e-value cutoff, 1e-10; word size, 28; match/mismatch scores, 1/−2; and gap costs, linear). The following sequence repositories were used with particular attention: *L. luteus*, http://www.cgna.cl/lupinus (project PRJNA170318, archive SRX159101); *L. albus*, http://comparative-legumes.org (gene index LAGI 1.0); and *L. angustifolius* (project PRJNA248164, archive GBRP00000000). Gene prediction in BACs was performed using FGENESH (Salamov and Solovyev 2000). Results of functional annotation were subsequently used for gene density (genes/kbp) calculation.

SSR Finder (http://www.fresnostate.edu/ssrfinder/) was used to identify single-sequence repeats (SSRs) with the following parameters: motif length, 2–8; minimum number of repeats, 2; and allowed nucleotide substitution. Repeats differing by reading frames (e.g., AG vs. GA) or reverse-complement reading (e.g., AG vs. CT) were clustered.

### Alignment to the draft *L. angustifolius* genome sequence

BESs were used to screen the collection of *L. angustifolius* whole-genome shotgun contigs and scaffolds (NCBI Project No. PRJNA179231, assembly version GCA_000338175.1). In the first step, BESs were aligned to the sequences deposited under accessions KB405099–KB441797; then, remaining unassigned BESs were aligned to sequences AOCW01000001–AOCW01191454, because these datasets do not completely overlap (KBs represent superscaffolds assembled from AOCWs; however, some AOCWs remained unassembled and do not exist in KB dataset). The BLAST algorithm was optimized for highly similar sequences (word size, 28; match/mismatch scores, 1/−2; and gap costs, linear), and one match with the highest score was selected. BES-producing alignments of sequence identity value above 95 % were checked for the possibility of base calling errors at mismatch sites, and in case of positive verification, appropriate scaffolds were considered as assigned.

### Microsyntenic region analysis

In order to identify microsyntenic regions of narrow-leafed lupin and nine Fabaceae species, *L. angustifolius* BAC and scaffold sequences with repetitive content were masked by RepeatMasker and Censor (Kohany et al. 2006) and subjected to comparative mapping. The following genome sequences were used: *Arachis duranensis* (Peanut Genome Project accession V14167, http://www.peanutbase.org), *A. ipaensis* (Peanut Genome Project accession K30076, http://www.peanutbase.org), *Cajanus cajan* (Varshney et al. 2012) (project PRJNA72815, v1.0), *Cicer arietinum* (Varshney et al. 2013) (v1.0 unmasked, http://comparative-legumes.org), *G. max* (Schmutz et al. 2010) (JGI v1.1 unmasked, http://www.phytozome.net), *L. japonicus* (Sato et al. 2008) (v2.5 unmasked, http://www.kazusa.or.jp), *M. truncatula* (Young et al. 2011) (strain A17, JCVI v4.0 unmasked, http://www.jcvi.org/medicago/), *P. vulgaris* (v0.9, DOE-JGI, and USDA-NIFA; http://www.phytozome.net), and *Vigna radiata* (Kang et al. 2014) (GenBank/EMBL/DDBJ accession JJMO00000000). The CoGe BLAST algorithm (Lyons et al. 2008) was used to perform sequence similarity analyses with the following parameters: e-value cutoff, 1e-20; word size, 8; gap existence cost, 5; gap elongation cost, 2; and nucleotide match/mismatch scores, 1/−2. Microsyntenic blocks were visualized using the Web-based Genome Synteny Viewer (Revanna et al. 2011) and Circos (Krzywinski et al. 2009).

### Molecular marker generation and genetic mapping

Primers for molecular markers amplification were designed based on BESs with the use of Primer3 Plus software (Untergasser et al. 2007). To identify polymorphic loci, PCR amplification was carried out with DNA isolated from parental lines of the *L. angustifolius* mapping population, 83A:476 (D = domesticated) and P27255 (W = wild). PCR products were visualized by 2 % agarose gel electrophoresis, purified with QIAquick PCR Purification Kit (Qiagen, Hilden, Germany), and sequenced on the ABIPRISM 3130 XL Genetic Analyzer (Applied Biosystems, Foster City, USA). Single-nucleotide polymorphisms (SNPs) were detected by the Cleaved Amplified Polymorphic Sequence (CAPS) approach (Konieczny and Ausubel 1993). Sequence differences such as sequence length polymorphism were performed by allele-specific PCR (AS-PCR) with primers anchored in polymorphic sites. Derived Cleaved Amplified Polymorphic Sequence (dCAPS) method, based on the use of mismatch PCR primers to introduce a restriction site into the polymorphic locus, was also used (Neff et al. 1998). Marker segregation was tested and the calculation of the chi-squared (*χ*^2^) for Mendelian segregation in *F*_8_ recombinant inbreed lines was done using the following expected segregation ratios: 0.4961 (maternal), 0.4961 (paternal), and 0.0078 (heterozygote). Probability calculation was based on *χ*^2^ and 2 *df*. The Map Manager QTXb20 freeware was used to localize new markers on the *L. angustifolius* genetic map (Kroc et al. 2014; Kamphuis et al. 2015). Graphic illustration of linkage groups was performed using MapChart (Voorrips 2002) software.

### Molecular probes for fluorescence in situ hybridization

#### BAC probes

BAC DNA was isolated using QIAprep Spin Miniprep Kit (Qiagen, Hilden, Germany) according to the protocol obtained by Farrar and Donnison (2007) and labeled with digoxigenin-11-dUTP and/or tetramethylrhodamine-5-dUTP (Roche Diagnostics, Basel, Switzerland) by nick translation, as described by Jenkins and Hasterok (2007).

#### rDNA probes

The wheat (*T. aestivum* var. Chinese spring) clone pTa794 (Gerlach and Dyer 1980) and *A. thaliana* 2.3-kb *Cla*I subclone (Unfried and Gruendler 1990) were used as 5S rDNA and 25S subunit of 45S rDNA reference sequences, respectively. rDNA probes as well as narrow-leafed lupin BAC clone 072O21 carrying 45S rDNA sequence (Książkiewicz et al. 2013) were labeled with digoxigenin-11-dUTP and/or tetramethyl-rhodamine-5-dUTP by nick translation (Roche Diagnostics, Basel, Switzerland).

*L. angustifolius* sequence 120E23_5 (GF110967.1) annotated as 5S rDNA (Książkiewicz et al. 2013) was labeled with tetramethyl-rhodamine-5-dUTP (Roche Diagnostics, Basel, Switzerland) by PCR according to Hasterok et al. (2004).

#### Oligonucleotide probes

To develop SSR-based probes, the particular motifs of sequences were synthesized and 5′ tailed, (ATCCTC)_3_, (GATAG)_3_, and (AGG)_5_ with digoxigenin-11-dUTP and (AG)_8_, (CTCC)_4_, and (CC)_8_ with biotin (Sigma Aldrich, St. Louis, USA).

### Chromosome preparation

Squash preparations of mitotic chromosomes of *L. angustifolius*, *L. luteus*, and *L. albus* as well as of *T. aestivum* and *N. tabacum* were prepared from the root meristems according to the published protocols (Leśniewska et al. 2011; Kwiatek et al. 2013). Enzymatic maceration of fixed material was performed (40 % pectinase (Sigma Aldrich, St. Louis, USA), 3 % cellulase (Sigma Aldrich, St. Louis, USA), and 1.5 % “Onozuka” cellulase (Serva Electrophoresis, Heidelberg, Germany). Dissected meristematic tissues were squashed on alcohol-cleaned slides in 60 % acetic acid. The quality of slides was checked under a phase-contrast microscope BX41 (Olympus, Tokyo, Japan). The slides were stored in −20 °C or directly used for FISH.

### FISH procedure

FISH procedure was carried out according to the protocol optimized for *L. angustifolius* (Leśniewska et al. 2011). Immunodetection of digoxigenin-labeled DNA probes was conducted with FITC-conjugated anti-digoxigenin primary antibodies (Roche Diagnostics, Basel, Switzerland), whereas biotin was labeled with streptavidin-Cy3 (Sigma Aldrich, St. Louis, USA). Chromosomes were counterstained with 2 μg/mL DAPI (Sigma Aldrich, St. Louis, USA) in Vectashield antifade mounting medium (Vector Laboratories, Burlingame, USA). Slides were analyzed with an epifluorescence microscope BX60 (Olympus, Tokyo, Japan) using the Cell_F software. The images of FISH signals and stained chromosomes were captured using a CCD monochromatic camera and superimposed using Micrografx (Corel, Ottawa, Canada) Picture Publisher 8 software.

## Results

### BAC clone characterization

To increase the chance of selecting single-locus BACs, which are required for integration of *L. angustifolius* genetic and cytogenetic maps, the sequence-specific probes used for BAC library screening were anchored in the sequences of four genes involved in nitrogen fixation, a process that is specific to legume plants, *ENOD40*, *NOD26*, *NOD45*, and *AAT*-*P2*. Altogether, 42 BAC clones yielded positive hybridization signals and were divided into independent libraries. The *ENOD40* library contained 21 BACs, the NOD26 library contained 5 BACs, the *NOD45* library contained 9 BACs, and the *AAT*-*P2* library contained 7 BACs. However, after Sanger sequencing with probe-specific primers, only 16 BACs were confirmed to carry the *ENOD40* sequence, and 5, 8, and 5 BACs were confirmed to carry the *NOD26*, *NOD45*, and *AAT*-*P2* sequences, respectively.

The BAC clones were grouped within the four libraries based on their sequence polymorphism determined by sequencing with gene-specific primers. Fourteen of the BAC clones that carried the *ENOD40* gene sequence shared 100 % nucleotide identity in the amplified sequences and were annotated as carrying *ENOD40*-*variant1* (Table 1). Two representative clones from this group were selected for in-depth studies, 008O20 and 131P18. The other two BAC clones that carried the *ENOD40* gene sequence, 059F07 and 068H10, were annotated as carrying *ENOD40*-*variant2* and *ENOD40*-*variant3*, respectively (Table 1). Likewise, the *NOD26* BAC clones were divided into the following three groups: clones 051D03 and 127N17 carrying *NOD26*-*variant1*, clone 087F06 carrying *NOD26*-*variant2*, and clones 123D01 and 066I06 carrying *NOD26*-*variant3* (Table 1). The *NOD45* BAC clones formed one homologous group carrying one gene variant. The *AAT*-*P2* BAC clones also formed one homologous group carrying one gene variant (Table 1).

**Table 1:** BAC clone characterization including BAC-FISH signal, genetic localization, and anchoring to scaffolds

BAC clone	Target gene	Verification by sequencing with target gene-based primers	Target gene sequence variant	FISH signal	NLL	Assigned sequence (BAC or scaffold)	Synteny (based on assigned sequence)
002B02	Random	−	−	Single	4	KB439932.1	+
004L17	*NOD45*	+	1	Single	−	−	−
008A17	*NOD45*	+	1	Single	−	138H12	+
008O20	*ENOD40*	+	1	Single	17	131P18	+
010I16	*ENOD40*	−	−	Repetitive	−	−	−
018I03	*ENOD40*	+	1	Single	−	131P18	+
020A06	Random	NI	NI	Single	11	AOCW01152381.1, KB427843.1	−
024K15	*ENOD40*	+	1	Repetitive	−	−	−
026B19	*AAT*-*P2*	+	1	Single	−	040A13	+
030F02	Random	NI	NI	Single	11	KB424747.1, KB436872.1	−
033G07	*ENOD40*	+	1	Single	−	131P18	+
036L23	*GS1*	+	NI	Single	11	AOCW01050906.1, KB412904.1	+
038J06	*NOD45*	+	1	Single	9	138H12	+
040A13	*AAT*-*P2*	+	1	Single	−	040A13	+
047P22	*GS1*	+	NI	Single	4	KB430042.1, KB405354.1	+
051D03	*NOD26*	+	1	Single	6	127N17	+
056N03	Random	NI	NI	Single	11	KB427843.1, KB423413.1	−
058D23	*ENOD40*	+	1	Repetitive	−	−	−
058G22	*AAT*-*P2*	+	1	Single	−	−	−
059A05	*ENOD40*	+	1	Repetitive	−	−	−
059F07	*ENOD40*	+	2	Single	6	059F07	+
059J08	*GS1*	+	NI	Single	9	AOCW01158579.1, KB441622.1	+
061F03	*NOD45*	+	1	Single	9	138H12	+
064J15	*PEPC*	+	NI	Repetitive	13	−	−
066I06	*NOD26*	+	3	repetitive	−	−	−
067C07	*PEPC*	+	NI	Single	19	KB415203.1, KB435497.1	+
067J14	*NOD45*	+	1	Single	−	138H12	+
068H10	*ENOD40*	+	3	Single	18	068H10	+
070A03	*NOD45*	−	−	single	−	AOCW01153660.1, KB407058.1	−
076D23	*AS*	+	NI	Single	14	AOCW01093208.1, AOCW01130134.1	+
081H12	*ENOD40*	+	1	Repetitive	−	−	−
083F23	*PEPC*	+	NI	Single	19	KB421397.1, KB435616.1	+
084A06	*AAT*-*P2*	−	NI	Single	3	KB431697.1	−
084H02	*ENOD40*	+	1	Repetitive	−	−	−
085K17	*ENOD40*	+	1	Repetitive	−	−	−
087F06	*NOD26*	+	2	Single	3	087F06	+
087N22	*GS1*	+	NI	Single	16	KB421253.1	−
088H05	*ENOD40*	+	1	Repetitive	−	−	−
089G04	*ENOD40*	+	1	Repetitive	−	−	−
095N22	*ENOD40*	−	−	Repetitive	−	−	−
097D16	*ENOD40*	−	−	Single	11	KB437521.1, AOCW01159605.1	+
102A04	*ENOD40*	−	−	Single	13	102A04	+
103E24	*ENOD40*	−	−	Repetitive	−	−	−
114O14	*AAT*-*P2*	+	1	Single	3	040A13	+
122B05	*NOD45*	+	1	Single	−	138H12	+
123D01	*NOD26*	+	3	Repetitive	19	−	−
127N17	*NOD26*	+	1	Single	6	127N17	+
130E19	*ENOD40*	+	1	Repetitive	−	−	−
131K15	*PEPC*	+	NI	Single	5	KB410794.1, KB422665.1	+
131K22	*AAT*-*P2*	−	−	Single	4	KB423564.1, KB405354.1	+
131P18	*ENOD40*	+	1	Single	17	131P18	+
132P08	*ENOD40*	+	1	Single	−	131P18	+
137N08	*AAT*-*P2*	+	1	Single	3	040A13	+
138D07	*NOD45*	+	1	Single	−	138H12	+
138H12	*NOD45*	+	1	Single	9	138H12	+
103O20	Centromere	NI	NI	Centromere	−	−	−
134A23	Centromere	NI	NI	Centromere	−	−	−
072O21	rDNA	NI	NI	rDNA	16	−	−
102E23	rDNA	NI	NI	rDNA	2	−	−

*NLL* narrow-leafed lupin linkage group, *NI* data not included

To saturate the cytogenetic map of *L. angustifolius*, the set of 42 BAC clones was supplemented with 13 other BACs selected from the narrow-leafed lupin BAC library. These clones carry the sequences of genes that encode *AS*, *GS1*, and *PEPC*. We also analyzed two BAC clones that revealed centromere-specific FISH signals (103O20 and 134A23) as well as two clones previously characterized as carrying rDNA sequences (072O21 and 120E23) (Książkiewicz et al. 2015). Altogether, 59 BAC clones were selected for further analysis using cytogenetic and molecular approaches (Table 1).

### BES analysis

To obtain sequence anchors for molecular marker development and *L. angustifolius* genome sequence survey, a set of 57 BAC clones were BAC-end sequenced, yielding 104 BESs (GenBank accession numbers KU678228–KU678331) with an average length of 568 bp and GC content 34.3 %. The RepeatMasker annotation marked 3434 bp out of a total BES length of 59,047 bp (5.82 %) as repetitive elements. These repeats included long interspersed nuclear elements (LINEs; 0.41 %), Ty1/Copia (2.53 %) and Gypsy/DIRS1 (0.65 %) retrotransposons, and DNA transposons (0.67 %) as well as single and low complexity repeats (1.49 and 0.07 %, respectively). Predicted non-repetitive gene-coding sequences were annotated in 28 of the 3′ BAC ends and in 20 of the 5′ ends (Online resource 2).

### BAC clones as narrow-leafed lupin chromosome markers

FISH was performed using 59 BACs as the molecular probes. To identify cytogenetic chromosome markers, the BAC probes were subjected to numerous FISH reactions on metaphase mitotic chromosomes of *L. angustifolius*. Various combinations of BAC-FISH reactions were performed using two probes simultaneously. Forty BACs gave single-locus type signals and were considered as chromosome-specific markers.

**Fig. 1:**
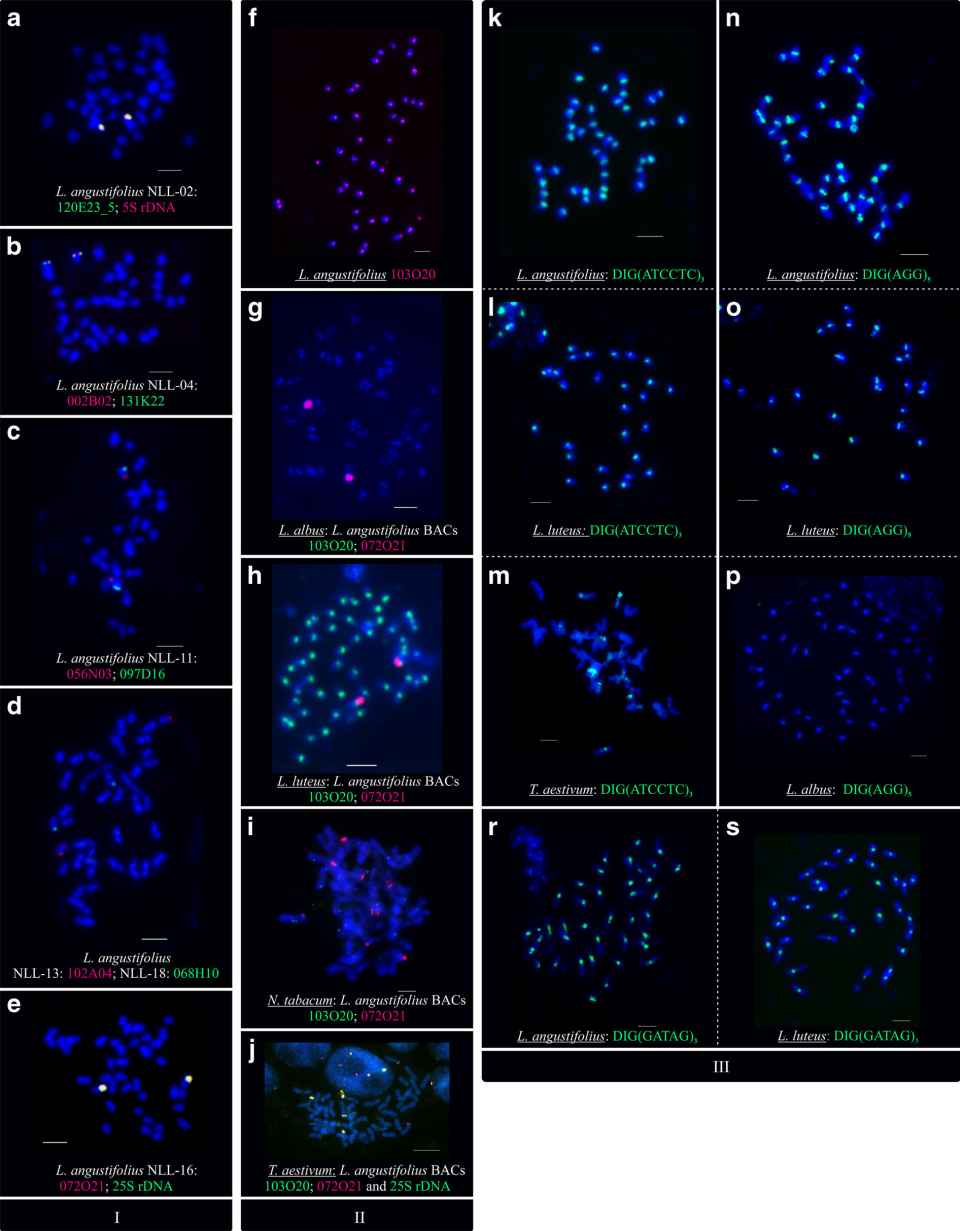
Localization of BAC-based chromosome-specific markers obtained as a useful toolbox for comprehensive analysis of *Lupinus angustifolius* genome and intergeneric comparative studies. I. Assignment of linkage groups to their corresponding chromosomes with BAC clone and rDNA sequences as FISH probes, **a** NLL-02: 120E23_5 (D), 5S rDNA (R); **b** NLL-04: 002B02 (R), 131K22 (D); **c** NLL_11: 056N03 (R), 097D16 (D); **d** NLL-13: 102A04 (R), NLL-18: 068H10 (D); and **e** NLL-16: 072O21 (R), 25SrDNA (D). II. Centromere-specific BAC clone 103O20 (D) as a tool for comparative analysis of centromere sequence divergence in plant chromosomes, **f** *L. angustifolius*, **g** *L. albus*, **h** *L. luteus*, **i** *N. tabacum*, and **j** *T. aestivum*. BAC clone 072O21 (R) carrying the 25S rDNA subunit of 45S rDNA was used as the BAC-FISH reaction control. III. Labeled SSR-anchored oligonucleotides as a tool for distinguishing three lupin species and *T. aestivum*, identification of **k**–**m** ATCCTC, **r**–**s** GATAG, and **n**–**p** AGG SSRs in chromosomes of *L. angustifolius*, *L. luteus*, *T. aestivum*, and *L. albus*. BAC clone DNA was labeled with tetramethylrhodamine-5-dUTP (R—*red* signal) or digoxigenin-11-dUPT (D—*green* signal). Overlapping clones produced *yellow* signals. Chromosomes were counterstained with DAPI. *Scale bar* = 5 μm (*L. angustifolius*, *L. albus*, and *L. luteus*) and 10 μm (*T. aestivum* and *N. tabacum*)

The following BACs, which originated from different libraries, localized in the same chromosome regions, but the regions differed from one library to the other: *ENOD40* (008O20, 018I03, 033G07, 131P18, and 132P08), *NOD26* (051D03 and 127N17), *NOD45* (004L17, 008A17, 038J06, 061F03, 067J14, 122B05, 138D07, and 138H12), and *AAT*-*P2* (026B19, 040A13, 058G22, 114O14, and 137N08). However, clones carrying different sequence variants, such as 008O20, 131P18 (*ENOD40*-*variant1*), 059F07 (*ENOD40*-*variant2*), and 068H10 (*ENOD40*-*variant3*), as well as 051D03, 127N17 (*NOD26*-*variant1*), and 087F06 (*NOD26*-*variant2*), were localized to different narrow-leafed lupin genome regions. The positions of BACs 123D01 and 066I06 (*NOD26*-*variant3*) in relation to the other two *NOD26* variants could not be determined because of the repetitive character of these clones. The localization of all chromosome-specific markers (both newly developed and already existing) was confirmed, allowing us to comprehensively verify physical linkage by direct visualization of reciprocal clone positions in narrow-leafed lupin chromosomes (Fig. 1a–e and Online resource 3).

The *L. angustifolius* 5S (120E23_5) and 25S (072O21) rDNAs were analyzed with reference rDNA sequences that are commonly used in FISH reactions. The mutual FISH mapping of 25S rDNA probe (*A. thaliana* 2.3-kb ClaI sub-clone) with BAC 072O21 carrying the fragment of 45S rDNA sequence (HR864186.1) (Książkiewicz et al. 2015) confirmed their localization in the same locus (Fig. 1e). Furthermore, the FISH also confirmed that the genetic marker 120E23_5 carrying 5S rDNA sequence (Książkiewicz et al. 2015) corresponded to the 5S rDNA (*T. aestivum* clone pTa794) locus (Fig. 1a). These clones/sequences constitute genetic and cytogenetic markers for rRNA genes in the narrow-leafed lupin genome.

Two *L. angustifolius* clones, 103O20 and 134A23, generated strong centromere-specific signals in all 20 chromosomes of the narrow-leafed lupin. Whole-BAC clones were used as molecular probes to follow their homology in four species, two in the *Lupinus* genus (*L. albus* and *L. luteus*), and two unrelated species (*N. tabacum* and *T. aestivum*). Centromere-specific BACs revealed strong hybridization signals in all centromeres in yellow lupin (*L. luteus*) chromosomes, whereas no specific signal was detected in white lupin (*L. albus*), tobacco, and wheat chromosomes (Fig. 1f–j).

Based on the results of the bioinformatic analysis of centromere-specific clone sequences (see “BAC clone sequencing and annotation” section), six SSR-specific FISH probes were designed and cytogenetically mapped. The SSR-FISH assay revealed clear centromere signals on *L. angustifolius* (Fig. 1k, n, r) and *L. luteus* (Fig. 1l, o, s) chromosomes for SSR probes (AGG)_5_ (Fig. 1n–p), (GATAG)_3_ (Fig. 1r, s), and (ATCCTC)_3_ (Fig. 1k–m) and very weak or no signal with probes (AG)_8_, (CTCC)_4_, and (CC)_8_. All SSR probes gave specific, but not centromere-specific, FISH hybridization patterns on *T. aestivum* chromosomes (Fig. 1m) and can serve as wheat chromosome markers (Table 2). No hybridization signal was detected for any of the SSR probes on *L. albus* (Fig. 1p) and *N. tabacum* chromosomes.

**Table 2:** Characterization of FISH signals detected for SSR-specific probes

SSR motif	*L. angustifolius* (2n = 40)	*L. luteus* (2n = 52)	*L. albus* (2n = 50)	*T. aestivum* (2n = 42)	*N. tabacum* (2n = 48)
DIG(ATCCTC)_3_	Centromere	Centromere	ND	Signals on several chromosomes	ND
DIG(GATAG)_3_	Centromere	Centromere	ND	Signals on several chromosomes, two dominant loci	ND
DIG(AGG)_5_	Centromere	Centromere	ND	Signals on several chromosomes	ND
Btn(AG)_8_	Very weak signals dispersed on all lupin chromosomes	ND	ND	Signals on two chromosome pairs	ND
Btn(CTCC)_4_	Very weak signals dispersed on all lupin chromosomes	ND	ND	Signals on three chromosomes	ND
Btn(CC)_8_	Very weak signals dispersed on all lupin chromosomes	ND	ND	Dominant signals on one chromosome pair, weak signals in two chromosome pairs	ND

*DIG* digoxigenin-labeled, *Btn* biotin-labeled, *ND* none detected

### BAC clone sequencing and annotation

BAC clones for sequencing were selected based on the following two criteria: type of BAC-FISH signal (single-locus clones were preferred) and target gene sequences represented. In total, 11 BAC clones were subjected to whole-insert sequencing, 9 carrying analyzed genes and 2 that were physically located in centromere regions of *L. angustifolius* chromosomes. Gene-specific BAC clones were selected from the following libraries: *ENOD40* (059F07, 068H10, and 131P18 carrying different gene sequence variants and 102A04 with no *ENOD40* sequence), *NOD26* (087F06, 123D01, and 127N17 carrying various gene copies), *NOD45* (138H12), and *AAT*-*P2* (040A13). The BAC clone lengths ranged from 14,351 to 108,291 bp that is consistent with Kasprzak et al. (2006), where the average insert size in the library was estimated to be ∼100,000 bp. GC content varied from 30.5 to 33.8 % (32.6 % on average). Repetitive content in BACs varied from 0.96 to 16.73 %. The most abundant were long terminal repeat (LTR) retrotransposons (Ty1/Copia and Gypsy/DIRS1) followed by DNA transposons. The average number of coding sequences within the BAC clones was established as 16 genes/100 kbp (Table 3 and Online resource 4).

**Table 3:** Characterization of the composition of BAC clone sequenced

BAC clone	Length (bp)	%GC	Percent of RE	RE type	No. of CDS
040A13	78,339	31.5	2.7	Ty1/Copia, simple repeats	11
059F07	96,808	32.8	4.28	Ty1/Copia, Gypsy/DIRS1, simple repeats	16
068H10	95,152	30.5	6.65	Ty1/Copia, simple repeats	10
087F06	92,290	32.6	1.51	DNA transposons, simple repeats	19
102A04	95,483	33.1	2.71	Simple repeats	19
123D01	94,935	33.4	16.73	Ty1/Copia, Gypsy/DIRS1, DNA transposons, simple repeats	19
127N17	14,351	33.8	0.96	Simple repeats	2
131P18	108,291	33.5	8.19	Ty1/Copia, Gypsy/DIRS1, simple repeats	16
138H12	93,952	33.0	2.31	L1/CIN4, simple repeats	14
103O20, 134A23	419,430^a^	54.3	92.5	Simple repeats	1

*REs* repeat elements, *CDS* coding sequence

^a^Total length of obtained multiple scaffolds

The assembly procedure of BAC clones that had generated BAC-FISH signals in centromere regions of *L. angustifolius* chromosomes resulted in generation of numerous small contigs for the clone 134A23 (954 sequences with minimum, mean, and maximum lengths of 183, 436, and 1159 nt, respectively) and one larger contig for 103O20 (3458 nt). These sequences displayed high GC (54.3 %) and SSR contents (92.5 %) and contained almost no gene-coding sequences (Table 3). The only annotated gene was “spindle pole body protein” (*pcp1*), which was shown to be involved in mitotic sister chromatid segregation (Flory et al. 2002).

SSR Finder was used to characterize more precisely the SSR motifs within centromere-BAC inserts. Clustering of repeats differing by reading frames or reverse-complement reading revealed two types of SSRs that were the most frequent, trinucleotides grouped as “GGA/AGG/GAG/CCT/CTC/TCC” and hexanucleotides grouped as “ATCCTC/TCCTCA/CCTCAT/CTCATC/TCATCC/CATCCT/AGGATG/GGATGA/GATGAG/ATGAGG/TGAGGA/GAGGAT.” The trinucleotide group constituted 23.0 % of the whole sequence, while the hexanucleotide group constituted 21.9 %. The other SRR motifs were grouped as follows: pentanucleotides “AGGAT/GGATA/GATAG/ATAGG/TAGGA/ATCCT/TCCTA/CCTAT/CTATC/TATCC” (9.2 %), dinucleotides “AG/GA/TC/CT” (8.6 %) and “CC/GG” (8.1 %), and tetranucleotides “CCCT/CCTC/CTCC/TCCC/AGGG/GAGG/GGAG/GGGA” (1.3 %). More complex internal sequence alignments showed that the vast majority of identified SSR motifs were ordered in longer structures that consisted of tandemly repeated units. Considering the average length of MiSeq v2 reads and the limitations of sequence assembly algorithms, we estimated that these structures were at least 200 bp in length. Annotation of the two longest reconstructed structures of 229 and 369 bp revealed that they were composed of two types of SSRs, the GGA and ATCCTC groups. In these two structures, the first SRR type was repeated 47 or 60 times and the second SRR type was repeated 17 or 27 times, respectively. However, considering the high probability of false positive overlapping of repeat-rich reads during sequence assembly, these numbers are only low-fidelity estimations. The raw BAC sequencing data has been deposited in GenBank (040A13: KU678217, 059F07: KU678219, 068H10: KU678221, 087F06: KU678218, 102A04: KU678222, 123D01: KU678220, 127N17: KU678223, 103O20: KU724479, 134A23: KU724480–KU725433, 131P18: KU678224, and 138H12: KU678225–KU678227).

### Anchoring BAC clones to the draft genome sequence

To reconstruct longer regions of the genome, the sequences of BAC clones 123D01, 138H12, and 131P18 as well as 28 BESs of the 14 clones carrying *ENOD40*-*variant1* were aligned to the scaffolds of the narrow-leafed lupin draft genome sequence (Yang et al. 2013). Overlapping scaffolds were identified for both ends of the studied clones. Based on these alignments, supercontigs carrying merged BAC and scaffold sequences were assembled. The BAC-based supercontigs consisted of 118,121 bp for 123D01, 171,311 bp for 138H12, and 295,679 bp for the set of 14 BACs and 10 scaffolds, including the 131P18 sequence. Then, to identify anchors of physical linkage and to group the remaining clones into contigs, all the BESs were aligned to sequenced BAC clones and to the new BAC-based supercontigs. Of the 104 BESs analyzed, 62 shared high-sequence identity (at least 97 % but mostly 99–100 %) with BAC clone and BAC-based supercontig sequences. Based on the BES mapping, the 131P18, 138H12, 040A13, 123D01, and 127N17 sequences were shown to represent contigs composed of 14, 7, 4, 2, and 2 clones, respectively. These results are concordant with BAC clone clustering based on sequencing from gene-specific primers. The alignment data are presented in Online resource 5.

To discover more nucleotide data related to analyzed cytogenetic landmarks, 33 BESs that did not match any of the BAC sequences but were from clones that yielded single-locus BAC-FISH signals were aligned to the scaffolds of the narrow-leafed lupin draft genome assembly (Yang et al. 2013). Lupin genome scaffolds were identified for all clones except 004L17 and 30 BESs from 16 clones aligned specifically to 27 scaffold sequences. The total length of the selected scaffolds was 462,589 bp, and the orientation of 26 of the scaffolds was determined by the location of two or more paired BESs.

Based on BES localization in the scaffolds, several other sets of physically overlapping clones were identified, one group of three clones (five scaffolds) and three groups of two clones (three to four scaffolds). Taking all the sequence data together, sequence representatives (whole inserts or genome scaffolds) were obtained for 37 of the 39 single-locus clones (Table 1). The list of BESs used for genome sequence screening is given in Online resource 6. Data on the assigned scaffolds including accession numbers, names, and lengths as well as basic statistics and coordinates of constructed alignments are presented in Online resource 7.

To supplement the information derived from whole-insert sequence analysis of chromosome-specific cytogenetic markers, the sequences of 32 scaffolds that were assigned to single-locus BAC clones (564,809 bp) were subjected to comprehensive in silico repeat content analysis. RepeatMasker and Censor screening revealed that the frequencies of interspersed repeats in these scaffolds varied from 0 to 61.75 %. A high prevalence of retrotransposons, particularly the Ty1/Copia and Gypsy/DIRS1 types, was observed. Transposon occupancy was negligible and the identified sequences originated from just four main families, Helitron, hAT, MuLE-MuDR, and CMC/EnSpm (Table 4). Repetitive elements constituted about 16 % of the scaffold sequences, but some single-locus clones revealed relatively high amounts of repetitive content, approximately 32 % for a contig of three BACs (020A06, 030F02, and 056N03) and approximately 33 % for clone 070A03. It should be noted that BAC clone sequences were not recovered in their entirety, because the lupin genome scaffolds were anchored to BAC ends and large gaps between them were to be expected. Consequently, the percentage values presented here should be considered as tentative estimates. Annotation data for the repeats, including sequence coordinates, *p* values, and repeat family/type assignment, are given in Online resource 8.

**Table 4:** Percentage of repetitive sequences identified in scaffolds that represent single-locus BAC clones

Type^a^	Content (%)
DNA/RC/Helitron	0.49
DNA/hAT	0.37
DNA/MuLE-MuDR	0.28
DNA/EnSpm/CACTA	0.16
DNA/other	0.18
Total transposons	1.48
R/LTR/Copia	6.71
R/LTR/Gypsy	4.48
R/LINE/L1	1.84
R/LINE/RTE	0.10
R/SINE/SINE2	0.08
R/LTR/ERV1	0.05
R/LTR/Pao	0.03
R/LINE/LOA	0.00
Total retrotransposons	13.29
Simple repeat	1.02
Total repeats	15.78

^a^Type, repetitive sequence type

**Fig. 2:**
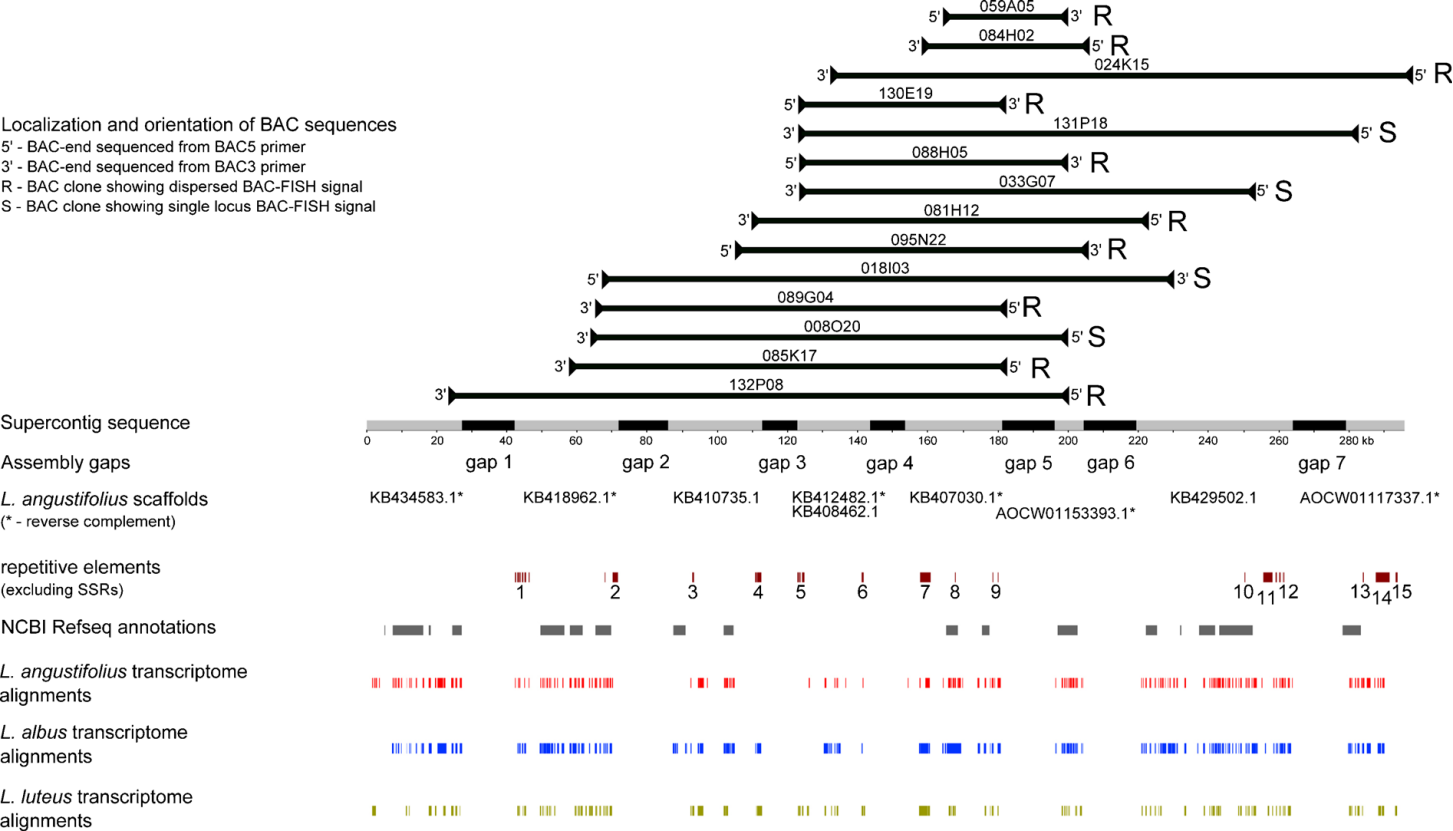
Graphical representation of the alignment of the supercontig encompassing 14 BAC clones carrying ENOD40-variant1 with NCBI RefSeq and *L. angustifolius*, *L. albus*, and *L. luteus* transcriptome sequences

The 131P18 supercontig, which carries *ENOD40*-*variant1* and consists of 10 scaffolds (14 clones), was aligned to the NCBI Refseq and transcriptome sequences of three lupin species using BLAST. The supercontig sequence aligned to numerous annotated *L. albus*, *L. luteus*, and *L. angustifolius* gene sequences as well as to cDNA contigs, as shown in Fig. 2 and Online resource 9. The alignments indicate that this supercontig contains a gene-rich region. However, the majority of BACs that formed this contig produced repetitive BAC-FISH signals that were dispersed over numerous chromosomes, and only four of them turned out to be single-locus probes. Because all the BES coordinates and clone orientations on the draft genome assembly were deciphered, we were able to analyze the type of BAC-FISH signals in relation to the sequence composition. Briefly, the presence of two large Copia segments or one Copia and one Gypsy segment in some of the BAC clones was enough to produce repetitive signals in BAC-FISH. Similarly, the presence of Helitron together with one large Copia element produced repetitive BAC-FISH signals. On the contrary, the presence of LINE/L1 or hAT repeats was not related to the type of BAC-FISH signal produced (Online resource 10).

### Genetic mapping

To assign selected BAC clones to linkage groups of the narrow-leafed lupin genetic map, we developed molecular markers to integrate the chromosomal and genetic maps. For primer design, we used 81 sequences derived from 39 single-locus BACs and 3 from repetitive sequences carrying target gene sequences.

Specific PCR products, amplified on a template of DNA isolated from parental lines of the mapping population (83A476 and P27255), were obtained for all the BES-derived and gene-specific primer pairs. Sequence polymorphism between parental lines was identified for 32 primer pairs, and molecular markers were designed as follows: 22 by CAPS, 6 by AS-PCR, and 2 by dCAPS (Online resource 11). The analyzed sequences often exhibited more than one difference in their nucleotide composition. Some SNP-derived markers had additional length polymorphisms in the PCR products (008O20_5_3, 030F02_3, 059J08_3, 067C07_2, and 114O14_3). The obtained logarithm of the odds (LOD) scores of mapped markers varied from 4.6 to 20.6. Statistically significant deviation from the Mendelian segregation ratio (*p* value <0.05) was observed for five markers, namely 008O20_5_3, 020A06_3, 051D03_5, 097D16_3, and 131P18_3.

The studied *ENOD40*-*variant1* sequence was assigned to narrow-leafed lupin linkage group 17 (NLL-17) based on BES markers 008O20_5 and 131P18_3. Molecular markers 059F07_3 and 068H10_5 localized *ENOD40*-*variant2* and *ENOD40*-*variant3* in NLL-06 and NLL-18, respectively. Similarly, the *NOD26* sequence variants were genetically mapped and assigned to three linkage groups (051D03 and 127N17 to NLL-06, 087F06 to NLL-03, and 123D01 to NLL-19). *AAT*-*P2* molecular markers were anchored within BAC clones 114O14 and 137N08, which localized these sequences in *L. angustifolius* chromosome 3 (Lang 03). The supplementary, randomly selected BAC clones were genetically mapped and complemented the set of chromosome-specific markers. The *AS* gene was mapped to NLL-14 based on the clone 076D23 sequence. BAC 131K15, which contains the sequence of the *PEPC*-*variant*, was assigned to NLL-05, whereas clones 067C07 and 083F23 were assigned to NLL-19. The *GS1* genes represented by clones 036L23, 047P22, 059J08, and 087N22 were assigned to NLL-04, NLL-09, NLL-11, and NLL-16, respectively. Moreover, the physical linkage of clones in five contigs was confirmed by genetic mapping of BES-derived markers, two in NLL-03 (114O14_3 and 137N08_5), three in NLL-09 (038J06_3, 061F03_5, and 138H12_5), three in NLL-11 (020A06_3, 056N03_3, and 030F02_3), two in NLL-17 (008O20_5 and 131P18_5), and two in NLL-19 (067C07_3 and 083F23_3). Molecular marker sequences have been deposited in GenBank under accession numbers KU662037–KU662091.

**Fig. 3:**
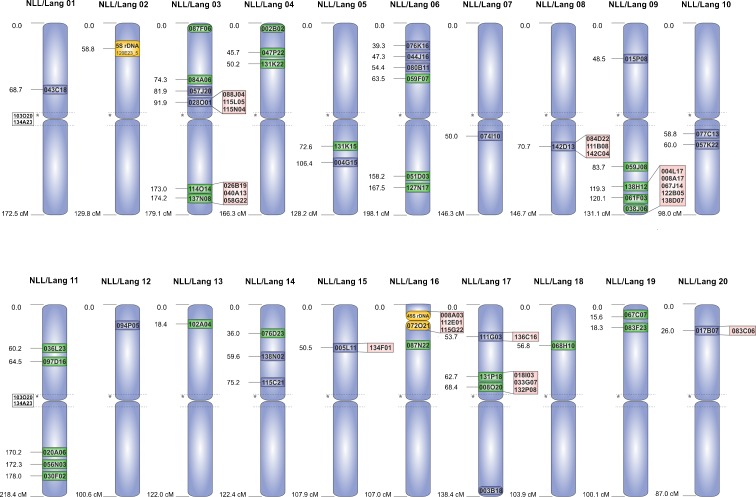
Schematic representation of integrated linkage groups and chromosomes of *Lupinus angustifolius*. BAC clones used as parallel genetic and cytogenetic markers are shown as *green* (newly developed markers), *blue* (previously described markers), or *yellow* (rDNA) and positioned according to the genetic distance (in cM). The BAC clones established only as chromosome-specific markers are displayed in *red* and marked outside the chromosomes (*lines* correspond to defined chromosome loci)

All 32 new markers, including 5S rDNA and 45S rDNA, were distributed into 13 linkage groups of *L. angustifolius* (Online resource 12). Eleven markers derived from single-locus BAC clones allowed us to assign all remaining unassigned linkage groups to chromosomes, thereby completing map integration for *L. angustifolius* (Fig. 3).

### Cross-genus microsynteny of single-locus BAC clones

**Fig. 4:**
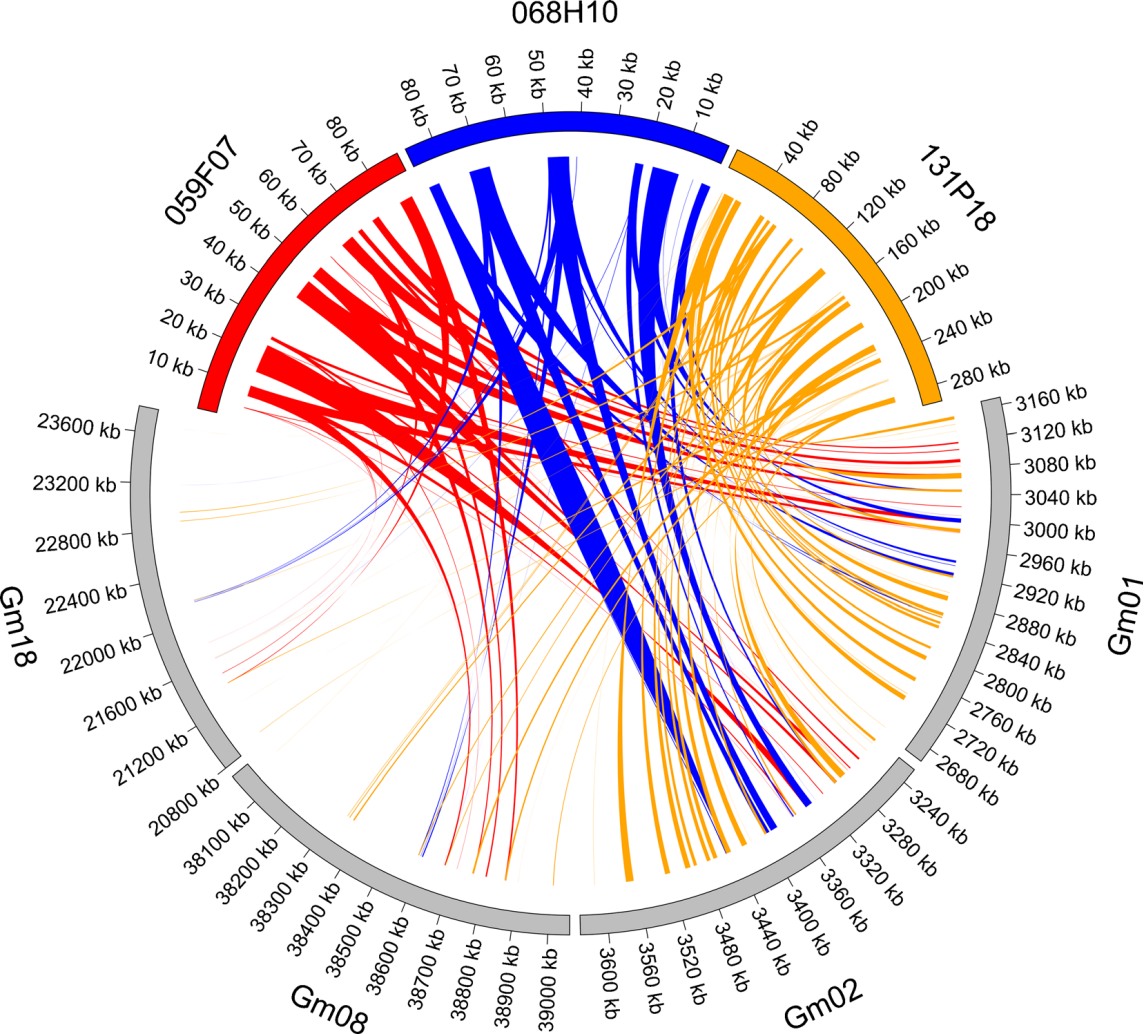
Legume cross-genus microsyntenic patterns revealed for narrow-leafed lupin *ENOD 40* clones (059F07, 068H10, 131P18) and *Glycine max* chromosomes (Gm01, Gm02, Gm08, Gm18). Ribbons visualize homologous links identified by DNA sequence similarity. The chromosomes and BAC clones are not drawn to scale

BAC clone sequences that yielded single-locus FISH signals on *L. angustifolius* metaphase chromosomes and scaffolds anchored to these clones were subjected to comparative analysis, to determine if these BACs constitute cytogenetic landmarks of regions with conserved structure and shared synteny among several legume species. Syntenic patterns were observed for 23 sequences, representing (by physical linkage) 31 single-locus BAC clones. Data on all identified links of collinearity are provided in Online resource 13, and visualization of the most conserved syntenic blocks is presented in Fig. 4 and Online resource 14a–g.

Scaffolds anchored to *AS* clone 076D23 revealed syntenic links to regions carrying the *AS* gene on *A. duranensis* chromosome 10 (Ad10), *A. ipaensis* chromosome 10 (Ai10), *C. arietinum* chromosome 5 (Ca5), *C. cajan* chromosome 1 (Cc1), *G. max* chromosome 18 (Gm18), *M. truncatula* chromosome 3 (Mt3), *P. vulgaris* chromosome 1 (Pv1), and *V. radiata* chromosome 2 (Vr2) as well as to regions lacking the *AS* gene on Ad3, Ai3, Ca6, Gm11, Gm5, Gm8, Mt8, Pv2, and Vr7 chromosomes and two Cc scaffolds. Such a pattern suggests ancient duplication in these species, followed by loss of excessive gene copies.

Scaffolds mapped to BAC clones 036L23, 047P22, 059J08, and 131K22 carrying the *GS* gene shared synteny with loci in the genomes of other legume plants, all of which carried at least one homologous copy of the *GS* gene. These observations provide clear evidence of ancient duplications that did not result in further elimination of additional gene copies. However, the overall pattern is more complex, because scaffolds anchored to 036L23 shared very well preserved synteny with regions lacking an annotated *GS* gene copy.

The set of scaffolds matching clones carrying *PEPC* had the most complex pattern of collinearity links, reflecting the numerous local duplications in the analyzed legume species. Two types of syntenic relationships were observed, to regions carrying a *PEPC* gene (scaffolds mapped to clones 083F23 and 131K15 or 083F23 itself) and to regions lacking a *PEPC* gene (scaffolds mapped to 067C07).

Three clones carrying an *ENOD40* sequence showed links of conserved collinearity to the same regions on Ad3, Ad5, Ai3, Ai5, Ca2, Cc6, Gm1, Gm2, Gm8, Lj2, Mt5, Pv2, and Vr11. Such a distribution of syntenic blocks evidenced well-conserved remnants of duplication in *Arachis* and *Glycine* genomes (Fig. 4). Moreover, additional loci were identified on Ca7, Gm18, and Mt8 for 131P18; on Vr_scaffold_206 for 059F07; and on Pv3 for 131P18 and 059F07. Because the ENOD40 protein has only 14 amino acids, of which only 5 are conserved, we failed to identify this protein in corresponding legume regions due to the high number of putative false positive alignments.

Two BAC clones possessing a *NOD26* gene, 087F06 and 127N17, revealed syntenic links to loci on Ad7, Ai8, Ca1, Cc11, Gm13, Gm15, Lj3, Mt2, Pv5, and Vr5. All these loci were annotated as “water-selective transport intrinsic membrane protein” or “major intrinsic protein,” believed to be synonymous to *NOD26* gene. Moreover, BAC clone 087F06 revealed synteny to loci on Ad9 and Ai9 annotated as “intrinsic protein,” as well as to loci on Gm7, Gm8, Pv10, and Vr9 that lacked an annotated *NOD26*/intrinsic protein homolog.

BAC clone 138H12 with a *NOD45* sequence showed conserved synteny to regions on chromosomes Ad1, Ad7, Ai1, Ai7, Ca2, Cc2, Gm14, Gm18, Gm2, Gm8, Lj2, Mt3, Mt5, Pv6, Pv8, and Vr6. However, no statistically significant alignment to a *NOD45* gene sequence was identified in those regions or in the whole-genome assemblies of all the analyzed legume species, indicating that *NOD45* is putatively a lupin-specific gene.

BAC clone 040A13 carrying a *AAT* gene showed well-preserved collinearity to regions annotated as AAT on Ad6, Ai6, Ca4, Cc_Scaffold137572, Gm14, Gm17, Lj5, Mt1, Pv1, and Vr6. It also had syntenic links to regions lacking an annotated *AAT* on Ca5, Gm4, Gm6, Mt3, Pv9, and Vr6. Duplicated regions had many local rearrangements, mostly insertions/deletions.

The set of clones showing single-locus signals on metaphase chromosomes also included BAC clones selected randomly from the library. Three of the “random” clones revealed distinct patterns of synteny, two indirectly by BES-anchored scaffolds 002B02 and 097D16 and one directly by whole-insert sequence 102A04. Such an observation connects with the results of the linkage and cytogenetic mapping because these random clones were mapped in different chromosomes and linkage groups of *L. angustifolius*.

## Discussion

### Integrated map of the *L. angustifolius* genome as a platform for genus-wide cytogenetic studies

Integrative genome mapping can generate essential information for the improvement of sequence assembly in whole-genome sequencing and can provide a foundation for comparative genomics and evolutionary studies (Ren et al. 2012). Such integrated maps should facilitate and accelerate the ongoing second stage of whole-genome assembly of the narrow-leafed lupin by resolving the order and physical positions of linked markers and evaluating the size of the remaining gaps. Moreover, an integrated genetic/physical map may help to greatly improve the ordering of the reference genome superscaffolds into chromosomal “pseudomolecules,” as was shown previously for potato (Sharma et al. 2013). Here, we report a consensus genome map for narrow-leafed lupin, which was achieved by combining cytogenetic and genetic mapping. On the integrated map, 71 BAC clones, and 5S rDNA and 25S subunits of 45S rDNA sequences as chromosome-specific cytogenetic markers, tagged all 20 linkage groups of *L. angustifolius* (Fig. 3). The observed distribution of molecular markers is disproportionate among all the lupin chromosomes; i.e., six anchoring points are available for narrow-leafed lupin linkage group 3 (NLL-03) and chromosome 3, whereas only one is available for NLL/Lang 01. Similar marker density on an integrated map for *P. vulgaris* has been reported (Fonseca et al. 2010). Nevertheless, the set of chromosome-specific BAC markers, together with rDNA sequences, characterizes the *L. angustifolius* karyotype and constitutes a useful tool for evolutionary studies within the *Lupinus* genus. The established platform of cytogenetic landmarks is sufficient to distinguish various chromosome regions including the determination of particular chromosome arms. The integrated narrow-leafed lupin map can be used to track chromosome rearrangements and their influence on genome evolution. Because special attention was paid to ensure that molecular markers were developed directly from single-locus BACs, the individual BACs applied as FISH probes corresponded to the same loci as the genetically mapped sequences. Such an approach is particularly important for species with small uniform chromosomes, particularly those that carry well-evidenced remnants of ancient whole-genome duplication or triplication (Kroc et al. 2014).

Map integration is useful for identifying genes/regions that confer important traits and for the development of sequence-defined markers for breeding programs, as was shown in watermelon (Ren et al. 2012) and in narrow-leafed lupin (Książkiewicz et al. 2015). We have previously assigned 13 linkage groups to chromosomes by 20 BAC clones (Książkiewicz et al. 2015; Przysiecka et al. 2015). Narrow-leafed lupin linkage group 12 (NLL-12) was assigned to the corresponding chromosome using BAC 094P05 as a molecular probe (Narożna 2014). In the present study, we have saturated the genetic map with 30 new molecular markers representing genomic regions located in the 13 *L. angustifolius* linkage groups and analyzed the data that were obtained about the localization of the *ENOD40*, *NOD26*, *NOD45*, *AAT*-*P2*, *AS*, *GS1*, and *PEPC* genes in these linkage groups. Genetic mapping followed by cytogenetic localization of single-locus BAC clones allowed us to assign six remaining linkage groups to corresponding chromosomes, thus complementing their reciprocal assignment in *L. angustifolius*. A distinctive feature of all but one single-locus BACs mapped in this study was their localization to distal chromosomal regions; only BAC 070A03 was localized close to the pericentromeric region. This observation is consistent with previous findings that gene density was generally higher in distal regions rather than other regions of chromosomes (Erayman et al. 2004). The BAC clone 070A03 sequence had various features that prevented its precise genetic mapping; therefore, this clone was not assigned to the narrow-leafed lupin genetic map.

Two *L. angustifolius* BACs, which in their BESs were annotated as containing 25S subunit of 45S and 5S rDNA sequences, were identified previously (Książkiewicz et al. 2015). The presence of rDNA sequences in these BACs was confirmed in the present study by cytogenetic co-localization of conserved reference sequences 5S and 25S with PCR products amplified using BAC DNA as a template and BAC, respectively. FISH analysis of these clones/rDNA markers revealed the cytogenetic localization of 5S rDNA and 18S–5.8S–25S (45S rDNA) rDNA loci in two distinct chromosome pairs. Together with BES-based genetic markers, these clones allowed two linkage groups to be assigned to chromosomes. The clone with 5S rDNA was mapped to one locus in NLL-02. The clone containing 25S subunit of 45S rDNA was mapped to NLL-16. This clone was reported previously as a marker for the secondary constriction of the chromosome and for the nucleolar organizer in *L. angustifolius*. The results of the present study corroborate previous data on rDNA in *L. angustifolius* cv. Sonet (Naganowska and Zielinska 2002; Kaczmarek et al. 2009). However, for *L. angustifolius* cv. Emir, Hajdera et al. (2003) found three additional minor 5S rDNA signals. It is worth noting that Old World lupins vary not only in chromosome number (2n = 32–52) and genome size (0.97–2.68 pg/2C) but also in number and distribution of rDNA loci (Naganowska et al. 2003; Naganowska and Zielinska 2004). Thus far, chromosomal localization of rDNA has been performed in numerous papilionoid species, *C. cajan* (Varshney et al. 2012), *L. japonicus* (Ohmido et al. 2010), *P. vulgaris* (Fonseca et al. 2010), *G. max* (Krishnan et al. 2001), *A. ipaensis* and *A. duranensis* (Seijo et al. 2004), *M. truncatula* (Abirached-Darmency et al. 2005), *C. arietinum* (Abbo et al. 1994), and *V. radiata* (Bortoleti et al. 2012), as well as *L. angustifolius* (Naganowska and Zielinska 2002; Hajdera et al. 2003; Kaczmarek et al. 2009). Nevertheless, to the best of our knowledge, the present study is the first to provide information on linkage mapping of rDNA loci in the genus *Lupinus*.

### AGG and GATAC repeats as species-specific markers of *L. angustifolius* and *L. luteus* centromeric and pericentromeric regions

Centromeres are easily recognized regions in chromosome morphology. Although their functions are conserved among all eukaryotes, their DNA sequences are highly diverse, even among closely related species (Ma et al. 2007b). Such diversity makes genetic mapping of centromeres a very challenging task. However, FISH offers the possibility of mapping centromeres cytogenetically. Two of the BAC clones (103O20 and 134A23) that were subjected to BAC-FISH yielded clear reproducible signals over the centromeres of all the *L. angustifolius* chromosomes. Bioinformatic analysis of these BACs revealed that centromeric and pericentromeric regions of narrow-leafed lupin consisted of SSRs ordered into two types of sequence blocks. The centromere-specific tandem repeats contained the trinucleotide and pentanucleotide SSRs AGG and GATAC, structured into long arrays. The presence of two distinct sequences formed by the two types of SSR repeats is surprising because most diploid species studied so far possess only a single centromeric satellite (Jiang et al. 2003). However, *G. max* centromeres have been reported to be composed of two distinct satellite repeats (GmCent-1 and GmCent-4) and retrotransposon-related sequences (GmCR) (Tek et al. 2010). Detailed analyses of centromeric DNA have been limited mainly to species within the Gramineae and Brassicaceae families. The rapid evolution of centromeric satellites has been correlated with the appearance of diverged and/or novel centromeric sequences in *O. sativa* and *A. thaliana* (Melters et al. 2013). Although research into centromeres in legume chromosomes is still in its infancy, centromeric satellites have been identified in several species; for example, a satellite Ljcen1 localized in *L. japonicus* and MtR3 in *M. truncatula* have been reported (Kulikova et al. 2004). Moreover, SSRs have been identified as a major constituent of centromeric DNA in legume species (Iwata et al. 2013). Knowledge on centromere structure and the composition of SSRs is still rudimentary; therefore, the presence of long clusters of SSRs in lupins needs further investigation.

The comparative cytogenetic mapping of BACs 103O20 and 134A23 by their cross hybridization to chromosomes of four other species revealed signals in centromeres of *L. luteus*. However, no centromere-specific pattern was detected in *L. albus* or the other two species, *N. tabacum* and *T. aestivum*; the only signals visible were over some chromosome arms. This result supports the supposition of a closer evolutionary relationship between *L. angustifolius* and *L. luteus* compared with *L. albus* (Ainouche and Bayer 1999). Moreover, the synthetic oligonucleotides used in the FISH experiments revealed the trinucleotide and pentanucleotide SSR sites in the centromeric regions of chromosomes and confirmed the BAC-based results for the relationships among the analyzed species. Additionally, differences in the intensity of SSR-FISH signals were observed for *L. angustifolius* and *L. luteus*. This result suggests that the centromeric sequences of narrow-leafed and yellow lupins may have the same qualitative SSR composition, but their amounts or distributions may differ. Thus, the present study provides an indication that centromere-specific satellites are very divergent in the *Lupinus* genus.

### *L. angustifolius* landmarks of conserved genome regions and tools for tracking ancient duplications in lupins

BACs that show single-locus signals on chromosomes have low repetitive sequence content and high exon density (Belarmino et al. 2012; Książkiewicz et al. 2013). The annotation of nine single-locus BAC sequences in the present study is in agreement with these findings. The gene to distance ratios (16 genes/100 kbp on average) are similar to values previously found for other *L. angustifolius* gene-rich regions, 17.9 genes/100 kb in three BACs spanning ∼263 kb (Książkiewicz et al. 2013) and 19.3 genes/100 kb in 017B07 clone of ∼109 kb (Książkiewicz et al. 2015). Those values are higher than an average gene density of 9.7 genes/100 kb derived from the draft genome assembly (57 806 genes over 598 Mb) (Yang et al. 2013). Average GC content in sequenced BAC clones converges with the genome assembly statistics (32.6 vs 33.6 %) (Yang et al. 2013). Moreover, this value is consistent with previously published data for sequenced *L. angustifolius* BAC clones from this library, 33.1 % of GC content in five clones counting ∼453 kb in total (Książkiewicz et al. 2013) and 33.5 % GC content in five clones spanning ∼452 kb (Książkiewicz et al. 2015). In our study, average GC content in BESs was higher than in sequenced BACs (34.3 vs 32.6 %). The survey of 13,985 BESs carrying 8.89 Mbp of sequence data also revealed moderately high GC content value (39 %) (Gao et al. 2011). Such an observation might be explained by the fact that BESs represent specific restriction site-associated regions. Therefore, BES-derived sequence statistics cannot be extrapolated to the whole genome. Repeat content in sequenced gene-rich BACs (0.96–16.73) is within the range observed for other gene-rich regions of the species (Książkiewicz et al. 2013, 2015) and corresponds with the value of 11.81 % calculated for large BES dataset (Gao et al. 2011). The observed value is significantly lower than the total repeat content based on the K-mer plot model, estimated as 50 % (Yang et al. 2013), which is consistent with well-known feature of gene-rich regions, namely, low repetitive element coverage.

The sequences of gene-rich regions obtained in the present study may serve as a useful tool for evolutionary studies. Bioinformatic analysis of single-locus clones complemented the BAC-FISH approach by comparative mapping between *L. angustifolius* and several representatives of the main papilionoid clades. Direct sequence analysis provides additional information on genome sequence rearrangements, while FISH allows them to be visualized in chromosomes (Sobreira et al. 2011). Here, comparative mapping showed that at least 31 of the 40 chromosome-specific BAC anchors developed in this study were landmarks of well-conserved regions with cross-genus microsynteny. Considering that the level of synteny (i.e., the number and length of syntenic regions) is correlated with the evolutionary distance (Kaufmann and Frishman 2014), this set of clones should facilitate heterologous cytogenetic mapping of *Lupinus* species that are known to vary considerably in genome size and chromosome numbers. Sequence similarity analysis and cytogenetic mapping revealed the presence of more than one copy of *ENOD40*, *NOD26*, *GS1*, and *PEPC* gene sequences in the *L. angustifolius* genome. Comprehensive analysis at the gene and chromosome levels successfully localized all the studied gene copies into lupin linkage groups and chromosomes. The genetic positions of *ENOD40*, *NOD26*, *PEPC*, and *GS1* in the genome support the hypothesis of ancient duplications, which contributed to the evolution of distinct copies of these genes. Distinct gene copies were also identified in other legume genomes, in the regions syntenic to *L. angustifolius* carrying *NOD26*, *PEPC*, and *GS1* sequences. This finding implies that some of these duplication events must have occurred very early in the divergence history of papilionoid lineages. Indeed, some of these copies could be considered as remnants of an ancient whole-genome duplication that occurred in the progenitor line of papilionoids, including the genistoids (e.g., *L. angustifolius*), dalbergioids (*Arachis* spp.), millettioids (*P. vulgaris*, *G. max*, *C. cajan*, *V. radiata*), galegoids (*M. truncatula*, *L. japonicus*, *C. arietinum*), and *Xanthocercis* and *Cladrastis* (Schlueter et al. 2004; Pfeil et al. 2005; Cannon et al. 2006, 2015; Bertioli et al. 2009). Lupin whole-genome duplication is believed to have occurred before the divergence of New World and Old World clades (Cannon et al. 2015; Przysiecka et al. 2015), whereas some legume whole-genome duplication events occurred in recent evolutionary times, such as 13 million years ago in *G. max* and several million years ago in *Arachis* (Schmutz et al. 2010; Cannon et al. 2015).

The availability of the *L. angustifolius* draft genome sequence (Yang et al. 2013) and reference sequences of nine other legume species, *A. ipaensis*, *A. duranensis*, *C. cajan* (Varshney et al. 2012), *C. arietinum* (Varshney et al. 2013), *G. max* (Schmutz et al. 2010), *L. japonicus* (Sato et al. 2008), *M. truncatula* (Young et al. 2011), *P. vulgaris* (Schmutz et al. 2014), and *V. radiata* (Kang et al. 2014), has contributed significantly to lupin molecular studies. Despite the *L. angustifolius* genome sequence still being fragmented into numerous short scaffolds, the assembly, together with gene-based linkage maps, has opened unprecedented possibilities for comparative mapping and exploitation of microsynteny-based strategies. In the narrow-leafed lupin, the synteny shared with other sequenced legume genomes was harnessed to select candidate gene families underlying the vernalization independence locus *Ku* that confers an early flowering phenotype (Nelson et al. 2006), as well as to track the evolutionary history of duplicated chalcone isomerase-like genes (Przysiecka et al. 2015). Progress on the integration of genetic and chromosomal maps of *L. angustifolius*, together with the analysis of cross-genus sequence collinearity, represents a state-of-the-art method of identifying clones that represent the most conserved and stable regions of the lupin genome. Two large repeat-free gene-rich regions from single-locus BAC clones 004G15 and 080B11 served as anchors for comparative DNA sequence analysis between the genomes of *L. angustifolius* and *G. max*, which revealed identical order and orientation of microsyntenic blocks in the corresponding regions (Książkiewicz et al. 2013). The comparative analysis of gene-rich regions represented by single-locus BAC clone 017B07 provided novel evidence for shared synteny and ancient duplications in five of the legume species studied (*M. truncatula*, *G. max*, *L. japonicus*, *P. vulgaris*, and *C. cajan*) (Książkiewicz et al. 2015). Moreover, it has been demonstrated that ancient duplication regions are conserved among the main clades of *Papilionoideae*, dalbergioids (*Arachis*), genistoids (*Lupinus*), millettioids (*Cajanus*, *Glycine*, *Phaseolus*), robinioids (*Lotus*), and the inverted repeat-lacking clade (*Medicago*) (Lin et al. 2010; McClean et al. 2010; Reinprecht et al. 2013; Książkiewicz et al. 2015). However, until now, a very limited number of *L. angustifolius* chromosome landmarks that expose shared synteny have been developed, five clones from NLL-03, two from NLL-15, and one from NLL-06 (Książkiewicz et al. 2013, 2015; Przysiecka et al. 2015). In the present study, a milestone improvement of this resource has been achieved and the obtained data may contribute greatly to future lupin evolution studies at the genome and chromosome levels.

## Electronic supplementary material

Below is the link to the electronic supplementary material.

Online resource 1 Characterization of sequence-defined probes used for *L. angustifolius* nuclear genome library screening. (PDF 112 kb)

Online resource 2 Annotation of predicted gene-coding sequences in 3′ and 5′ BAC-end sequences using BLASTX (e-value cutoff 1e-10). (XLSX 8 kb)

Online resource 3 Verification of the localization of chromosome-specific marker physical linkages by direct visualization of reciprocal clone distribution in narrow-leafed lupin chromosomes. Two BAC-FISH probes were used simultaneously. *Y* localization of BACs in the same narrow-leafed lupin chromosome; *N* BACs were assigned to different chromosomes. (XLSX 46 kb)

Online resource 4 Characterization and annotation of predicted gene-coding sequences in BAC clones using BLASTX. (XLSX 111 kb)

Online resource 5 BAC-end sequences assigned to sequenced BAC clones. (XLS 13 kb)

Online resource 6 BAC-end sequences used for screening the narrow-leafed lupin genome sequence. (XLS 46 kb)

Online resource 7 Results of the alignment of BAC-end sequences (BES) to the *Lupinus angustifolius* genome sequence using BLAST. (XLS 21 kb)

Online resource 8 Repetitive sequences identified in *Lupinus angustifolius* scaffolds assigned to BAC clones that yielded single-locus BAC-FISH signals on metaphase chromosomes of *L. angustifolius*. (XLS 15 kb)

Online resource 9 Alignment of the 131P18 supercontig, carrying ENOD40-variant1 and consisting of 10 scaffolds (14 clones), to NCBI RefSeq and *L. albus*, *L. luteus*, and *L. angustifolius* transcriptome sequences. (XLS 394 kb)

Online resource 10 Repetitive sequences identified in the 131P18 supercontig (131P18_14BACs). (XLS 29 kb)

Online resource 11 Details on BAC-end molecular markers that were developed to integrate the chromosomal and genetic maps. Segregation data for a *Lupinus angustifolius* recombinant inbreed line mapping population are included. (XLS 51 kb)

Online resource 12 Reference *Lupinus angustifolius* linkage map supplemented with 32 newly developed markers. (PDF 9483 kb)

Online resource 13 Legume cross-genus syntenic regions identified for clones established as single-locus BACs in narrow-leafed lupin metaphase chromosomes. (XLS 79 kb)

Online resource 14 Graphical representation of the most conserved syntenic blocks identified between **a** 131P18, **b** 059F07, **c** 068H10, **d** 087F06, **e** 102A04, **f** 127N17, and **g** 138H12 for nine legume species. (PDF 15,164 kb)

## Abbreviations

*AAT*-*P2*: Aspartate aminotransferase P2
Ad: *Arachis duranensis* chromosome
Ai: *Arachis ipaensis* chromosome
*AS*: Asparagine synthetase
AS-PCR: Allele-specific PCR
BAC: Bacterial artificial chromosome
BAC-FISH: Fluorescence in situ hybridization with BACs as molecular probes
BES: BAC-end sequence
Ca: *Cicer arietinum* chromosome
CAPS: Cleaved Amplified Polymorphic Sequence
Cc: *Cajanus cajan* chromosome
*ENOD40*: Early nodulin 40
FISH: Fluorescence in situ hybridization
Gm: *Glycine max* chromosome
*GS*: Glutamine synthetase
*GS1*: Cytosolic glutamine synthetase
Lang: Narrow-leafed lupin chromosome
LINE: Long interspersed nuclear elements
Lj: *Lotus japonicus* chromosome
LOD: Logarithm of the odds
Mt: *Medicago truncatula* chromosome
NLL: Narrow-leafed lupin linkage group
*NOD26*: Nodulin 26
*NOD45*: Nodulin 45
*PEPC*: Phosphoenolpyruvate carboxylase
Pv: *Phaseolus vulgaris* chromosome
SNPs: Single-nucleotide polymorphisms
SSR: Single sequence repeat
Vr: *Vigna radiata* chromosome
