# Supplementary material for: Integration of Lupinus angustifolius L. (narrow-leafed lupin) genome maps and comparative mapping within legumes
Source: Chromosome Res. 2016 May 11;24:355–78. doi: 10.1007/s10577-016-9526-8 (PMC4969343; doi:10.1007/s10577-016-9526-8)
Supplement: Supplementary file 1 — Characterization of sequence-defined probes used for L. angustifolius nuclear genome library screening. (PDF 112 kb) [file 10577_2016_9526_MOESM1_ESM.pdf]

Integration of *Lupinus angustifolius* L. (narrow-leafed lupin) genome maps and comparative mapping within legumes

Chromosome Research

Katarzyna Wyrwa, Michał Książkiewicz, Anna Szczepaniak, Karolina Susek, Jan Podkowiński, Barbara Naganowska

Institute of Plant Genetics of the Polish Academy of Sciences, Strzeszyńska 34, Poznań 60-479, Poland

Email: kwyr@igr.poznan.pl; Telephone: (+48 61) 65 50 217; Fax: (+48 61) 65 50 301

Online Resource 1. Characterization of sequence-defined probes used for *L. angustifolius* nuclear genome library screening.

Probe name	Accession number of probe sequence	Accession number of sequences used for primer design	Primers	Tm[a]	Probe length (bp)
ENOD40	KU678333	AF352375	F:CTCACACTCCACCACTTCCAACAGT<br>R:ACTGAAGAGAAACATATTCTAATCACCTGC	56°C	372
NOD26	KU678335	L12257	F: CWGYTKCWRTWGCTCATGCTTT<br>R: CAACRATGGAKCCRAGRAGCTG	54°C	200
NOD45	KU678332	L12388	F:AACACTATGATTTGGAAAGAAAGAGAATAGGTG<br>R:AACATTATACTAACTTCAAAGACTAGCATTGTGG	56°C	900
AAT-P2	KU678334	L29258	F: TACTCGAACCCACCTGTCCACG<br>R:TTTCAACCCCAAGGTTACAAAATTTCCTAAGG	58°C	904

[a]Tm, annealing temperature.
