# Supplementary material for: Integration of Lupinus angustifolius L. (narrow-leafed lupin) genome maps and comparative mapping within legumes
Source: Chromosome Res. 2016 May 11;24:355–78. doi: 10.1007/s10577-016-9526-8 (PMC4969343; doi:10.1007/s10577-016-9526-8)
Supplement: Supplementary file 12 — Reference Lupinus angustifolius linkage map supplemented with 32 newly developed markers. (PDF 9483 kb) [file 10577_2016_9526_MOESM12_ESM.pdf]

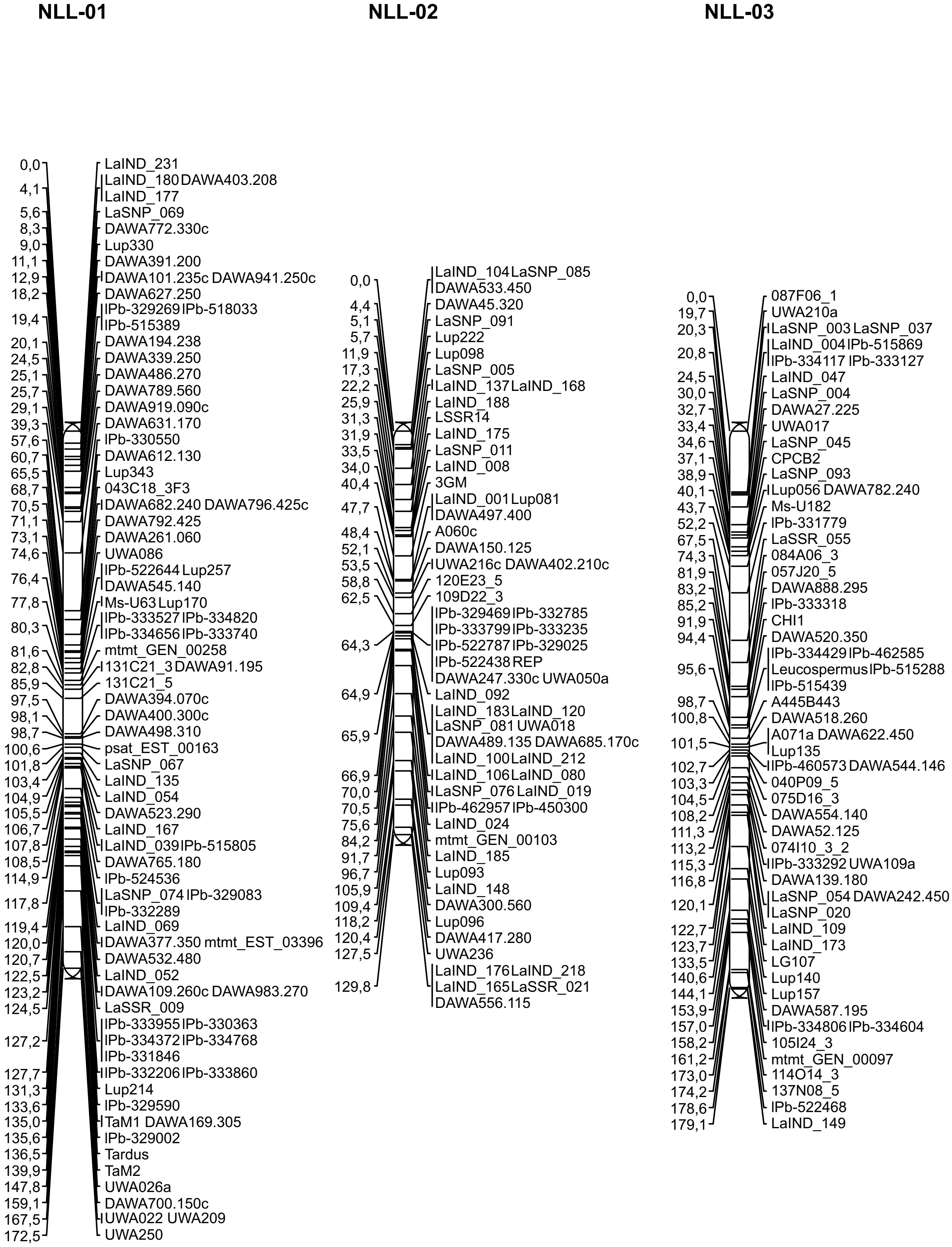

NLL-01
0,0 LaIND_231
4,1 LaIND_180 DAWA403.208
LaIND_177
5,6 LaSNP_069
8,3 DAWA772.330c
9,0 Lup330
11,1 DAWA391.200
12,9 DAWA101.235c DAWA941.250c
18,2 DAWA627.250
19,4 IPb-329269 IPb-518033
IPb-515389
20,1 DAWA194.238
24,5 DAWA339.250
25,1 DAWA486.270
25,7 DAWA789.560
29,1 DAWA919.090c
39,3 DAWA631.170
57,6 IPb-330550
60,7 DAWA612.130
65,5 Lup343
68,7 043C18_3F3
70,5 DAWA682.240 DAWA796.425c
71,1 DAWA792.425
73,1 DAWA261.060
74,6 UWA086
76,4 IPb-522644 Lup257
DAWA545.140
77,8 Ms-U63 Lup170
80,3 IPb-333527 IPb-334820
IPb-334656 IPb-333740
81,6 mtmt_GEN_00258
82,8 131C21_3 DAWA91.195
85,9 131C21_5
97,5 DAWA394.070c
98,1 DAWA400.300c
98,7 DAWA498.310
100,6 psat_EST_00163
101,8 LaSNP_067
103,4 LaIND_135
104,9 LaIND_054
105,5 DAWA523.290
106,7 LaIND_167
107,8 LaIND_039 IPb-515805
108,5 DAWA765.180
114,9 IPb-524536
117,8 LaSNP_074 IPb-329083
IPb-332289
119,4 LaIND_069
120,0 DAWA377.350 mtmt_EST_03396
120,7 DAWA532.480
122,5 LaIND_052
123,2 DAWA109.260c DAWA983.270
124,5 LaSSR_009
127,2 IPb-333955 IPb-330363
IPb-334372 IPb-334768
IPb-331846
127,7 IPb-332206 IPb-333860
131,3 Lup214
133,6 IPb-329590
135,0 TaM1 DAWA169.305
135,6 IPb-329002
136,5 Tardus
139,9 TaM2
147,8 UWA026a
159,1 DAWA700.150c
167,5 UWA022 UWA209
172,5 UWA250

NLL-02
0,0 LaIND_104 LaSNP_085
DAWA533.450
4,4 DAWA45.320
5,1 LaSNP_091
5,7 Lup222
11,9 Lup098
17,3 LaSNP_005
22,2 LaIND_137 LaIND_168
25,9 LaIND_188
31,3 LSSR14
31,9 LaIND_175
33,5 LaSNP_011
34,0 LaIND_008
40,4 3GM
47,7 LaIND_001 Lup081
DAWA497.400
48,4 A060c
52,1 DAWA150.125
53,5 UWA216c DAWA402.210c
58,8 120E23_5
62,5 109D22_3
64,3 IPb-329469 IPb-332785
IPb-333799 IPb-333235
IPb-522787 IPb-329025
IPb-522438 REP
DAWA247.330c UWA050a
64,9 LaIND_092
65,9 LaIND_183 LaIND_120
LaSNP_081 UWA018
DAWA489.135 DAWA685.170c
LaIND_100 LaIND_212
66,9 LaIND_106 LaIND_080
70,0 LaSNP_076 LaIND_019
70,5 IPb-462957 IPb-450300
75,6 LaIND_024
84,2 mtmt_GEN_00103
91,7 LaIND_185
96,7 Lup093
105,9 LaIND_148
109,4 DAWA300.560
118,2 Lup096
120,4 DAWA417.280
127,5 UWA236
129,8 LaIND_176 LaIND_218
LaIND_165 LaSSR_021
DAWA556.115

NLL-03
0,0 087F06_1
19,7 UWA210a
20,3 LaSNP_003 LaSNP_037
20,8 LaIND_004 IPb-515869
IPb-334117 IPb-333127
24,5 LaIND_047
30,0 LaSNP_004
32,7 DAWA27.225
33,4 UWA017
34,6 LaSNP_045
37,1 CPCB2
38,9 LaSNP_093
40,1 Lup056 DAWA782.240
43,7 Ms-U182
52,2 IPb-331779
67,5 LaSSR_055
74,3 084A06_3
81,9 057J20_5
83,2 DAWA888.295
85,2 IPb-333318
91,9 CHI1
94,4 DAWA520.350
95,6 IPb-334429 IPb-462585
Leucospermus IPb-515288
IPb-515439
98,7 A445B443
100,8 DAWA518.260
101,5 A071a DAWA622.450
Lup135
102,7 IPb-460573 DAWA544.146
103,3 040P09_5
104,5 075D16_3
108,2 DAWA554.140
111,3 DAWA52.125
113,2 074I10_3_2
115,3 IPb-333292 UWA109a
116,8 DAWA139.180
120,1 LaSNP_054 DAWA242.450
LaSNP_020
122,7 LaIND_109
123,7 LaIND_173
133,5 LG107
140,6 Lup140
144,1 Lup157
153,9 DAWA587.195
157,0 IPb-334806 IPb-334604
158,2 105I24_3
161,2 mtmt_GEN_00097
173,0 114O14_3
174,2 137N08_5
178,6 IPb-522468
179,1 LaIND_149

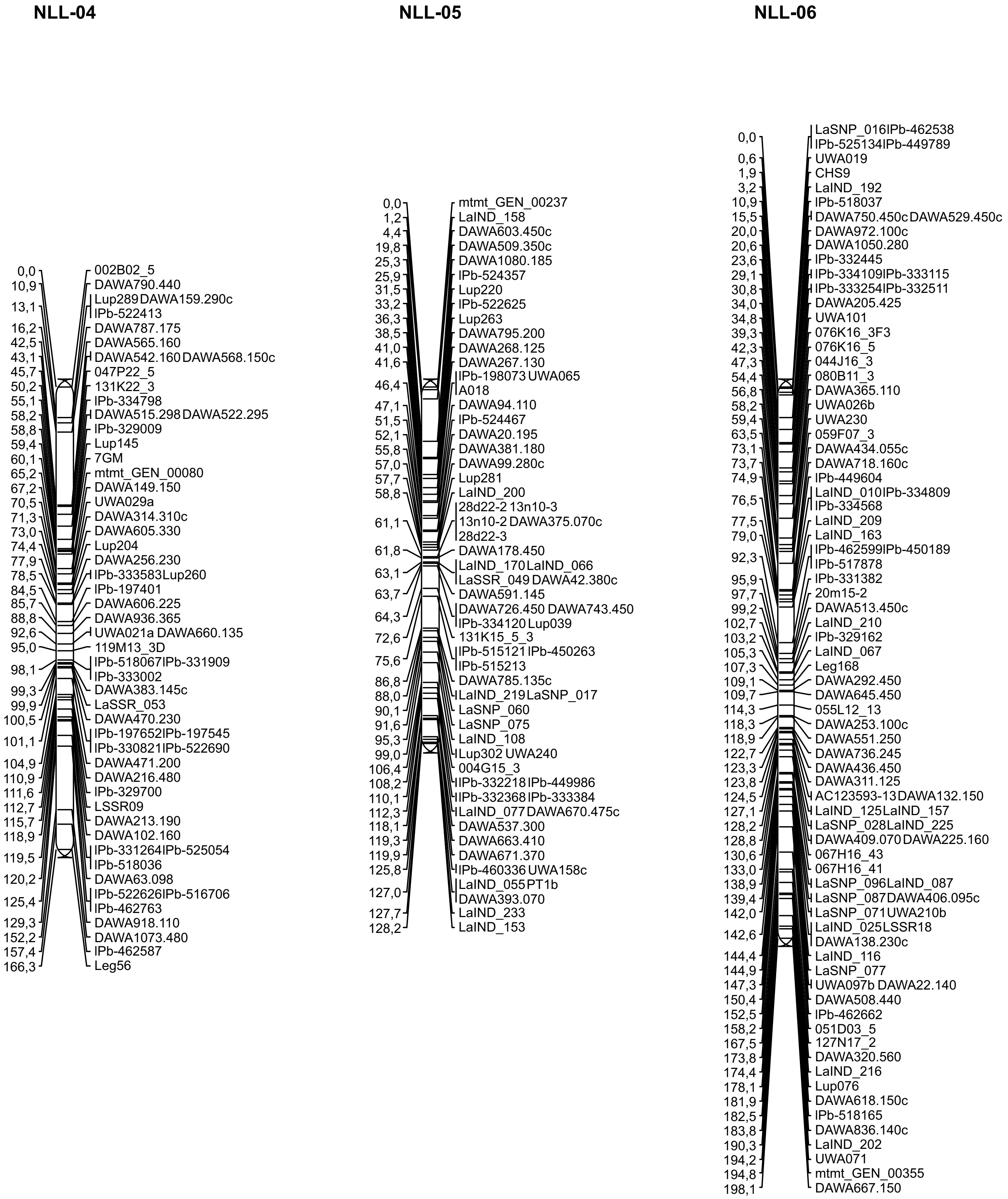
NLL-04
NLL-05
NLL-06
0,0 002B02_5
10,9 DAWA790.440
13,1 Lup289 DAWA159.290c
IPb-522413
16,2 DAWA787.175
42,5 DAWA565.160
43,1 DAWA542.160 DAWA568.150c
45,7 047P22_5
50,2 131K22_3
55,1 IPb-334798
58,2 DAWA515.298 DAWA522.295
58,8 IPb-329009
59,4 Lup145
60,1 7GM
65,2 mtmt_GEN_00080
67,2 DAWA149.150
70,5 UWA029a
71,3 DAWA314.310c
73,0 DAWA605.330
74,4 Lup204
77,9 DAWA256.230
78,5 IPb-333583 Lup260
84,5 IPb-197401
85,7 DAWA606.225
88,8 DAWA936.365
92,6 UWA021a DAWA660.135
95,0 119M13_3D
98,1 IPb-518067 IPb-331909
IPb-333002
99,3 DAWA383.145c
99,9 LaSSR_053
100,5 DAWA470.230
101,1 IPb-197652 IPb-197545
IPb-330821 IPb-522690
104,9 DAWA471.200
110,9 DAWA216.480
111,6 IPb-329700
112,7 LSSR09
115,7 DAWA213.190
118,9 DAWA102.160
119,5 IPb-331264 IPb-525054
IPb-518036
120,2 DAWA63.098
125,4 IPb-522626 IPb-516706
IPb-462763
129,3 DAWA918.110
152,2 DAWA1073.480
157,4 IPb-462587
166,3 Leg56
0,0 mtmt_GEN_00237
1,2 LaIND_158
4,4 DAWA603.450c
19,8 DAWA509.350c
25,3 DAWA1080.185
25,9 IPb-524357
31,5 Lup220
33,2 IPb-522625
36,3 Lup263
38,5 DAWA795.200
41,0 DAWA268.125
41,6 DAWA267.130
46,4 IPb-198073 UWA065
A018
47,1 DAWA94.110
51,5 IPb-524467
52,1 DAWA20.195
55,8 DAWA381.180
57,0 DAWA99.280c
57,7 Lup281
58,8 LaIND_200
61,1 28d22-2 13n10-3
13n10-2 DAWA375.070c
28d22-3
61,8 DAWA178.450
63,1 LaIND_170 LaIND_066
LaSSR_049 DAWA42.380c
63,7 DAWA591.145
64,3 DAWA726.450 DAWA743.450
IPb-334120 Lup039
72,6 131K15_5_3
75,6 IPb-515121 IPb-450263
IPb-515213
86,8 DAWA785.135c
88,0 LaIND_219 LaSNP_017
90,1 LaSNP_060
91,6 LaSNP_075
95,3 LaIND_108
99,0 Lup302 UWA240
106,4 004G15_3
108,2 IPb-332218 IPb-449986
110,1 IPb-332368 IPb-333384
112,3 LaIND_077 DAWA670.475c
118,1 DAWA537.300
119,3 DAWA663.410
119,9 DAWA671.370
125,8 IPb-460336 UWA158c
127,0 LaIND_055 PT1b
DAWA393.070
127,7 LaIND_233
128,2 LaIND_153
0,0 LaSNP_016 IPb-462538
IPb-525134 IPb-449789
0,6 UWA019
1,9 CHS9
3,2 LaIND_192
10,9 IPb-518037
15,5 DAWA750.450c DAWA529.450c
20,0 DAWA972.100c
20,6 DAWA1050.280
23,6 IPb-332445
29,1 IPb-334109 IPb-333115
30,8 IPb-333254 IPb-332511
34,0 DAWA205.425
34,8 UWA101
39,3 076K16_3F3
42,3 076K16_5
47,3 044J16_3
54,4 080B11_3
56,8 DAWA365.110
58,2 UWA026b
59,4 UWA230
63,5 059F07_3
73,1 DAWA434.055c
73,7 DAWA718.160c
74,9 IPb-449604
76,5 LaIND_010 IPb-334809
IPb-334568
77,5 LaIND_209
79,0 LaIND_163
92,3 IPb-462599 IPb-450189
IPb-517878
95,9 IPb-331382
97,7 20m15-2
99,2 DAWA513.450c
102,7 LaIND_210
103,2 IPb-329162
105,3 LaIND_067
107,3 Leg168
109,1 DAWA292.450
109,7 DAWA645.450
114,3 055L12_13
118,3 DAWA253.100c
118,9 DAWA551.250
122,7 DAWA736.245
123,3 DAWA436.450
123,8 DAWA311.125
124,5 AC123593-13 DAWA132.150
127,1 LaIND_125 LaIND_157
128,2 LaSNP_028 LaIND_225
128,8 DAWA409.070 DAWA225.160
130,6 067H16_43
133,0 067H16_41
138,9 LaSNP_096 LaIND_087
139,4 LaSNP_087 DAWA406.095c
142,0 LaSNP_071 UWA210b
142,6 LaIND_025 LSSR18
DAWA138.230c
144,4 LaIND_116
144,9 LaSNP_077
147,3 UWA097b DAWA22.140
150,4 DAWA508.440
152,5 IPb-462662
158,2 051D03_5
167,5 127N17_2
173,8 DAWA320.560
174,4 LaIND_216
178,1 Lup076
181,9 DAWA618.150c
182,5 IPb-518165
183,8 DAWA836.140c
190,3 LaIND_202
194,2 UWA071
194,8 mtmt_GEN_00355
198,1 DAWA667.150

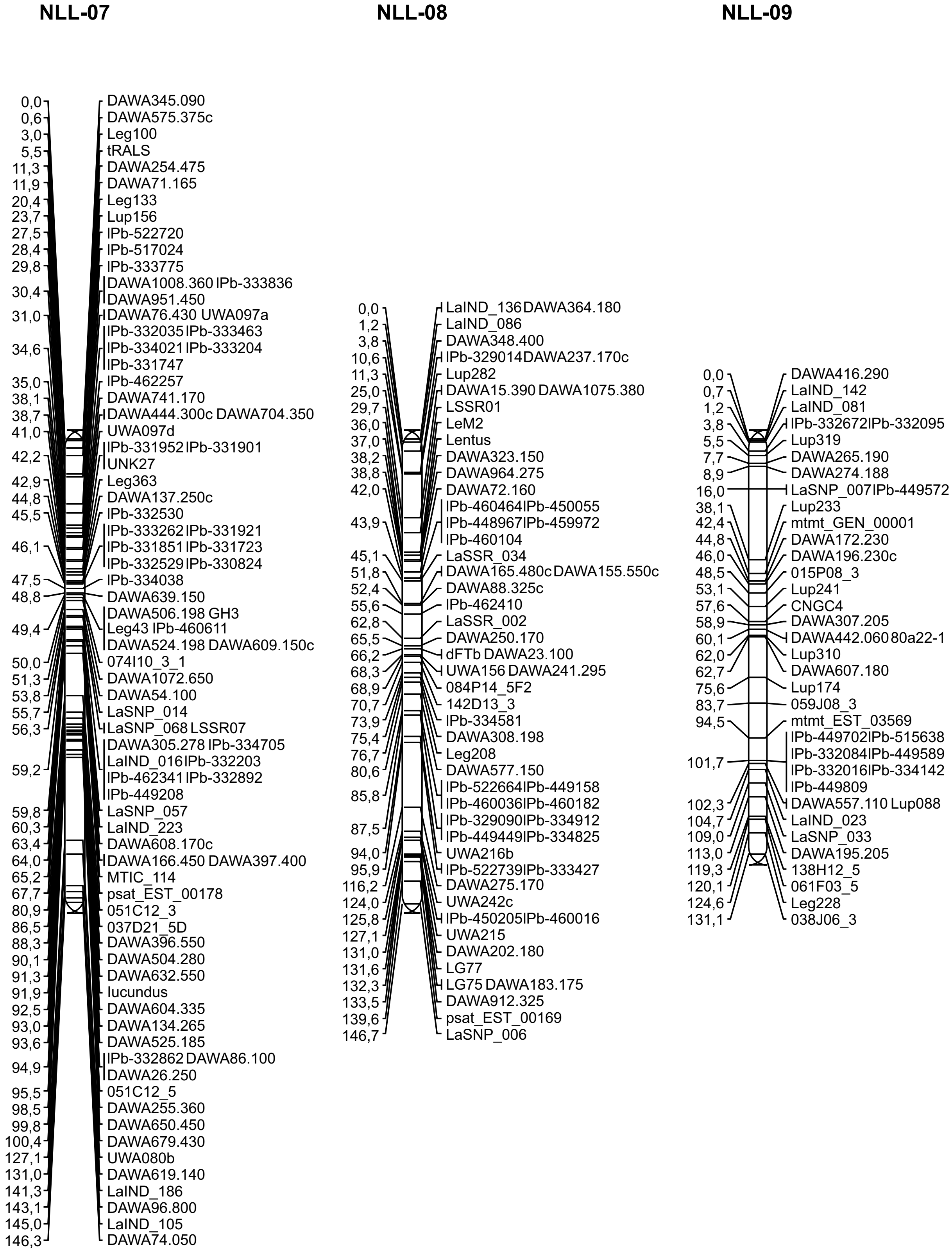

NLL-07
0,0 DAWA345.090
0,6 DAWA575.375c
3,0 Leg100
5,5 tRALS
11,3 DAWA254.475
11,9 DAWA71.165
20,4 Leg133
23,7 Lup156
27,5 IPb-522720
28,4 IPb-517024
29,8 IPb-333775
30,4 DAWA1008.360 IPb-333836 DAWA951.450
31,0 DAWA76.430 UWA097a
34,6 IPb-332035 IPb-333463 IPb-334021 IPb-333204 IPb-331747
35,0 IPb-462257
38,1 DAWA741.170
38,7 DAWA444.300c DAWA704.350
41,0 UWA097d
42,2 IPb-331952 IPb-331901 UNK27
42,9 Leg363
44,8 DAWA137.250c
45,5 IPb-332530
46,1 IPb-333262 IPb-331921 IPb-331851 IPb-331723 IPb-332529 IPb-330824
47,5 IPb-334038
48,8 DAWA639.150
49,4 DAWA506.198 GH3 Leg43 IPb-460611 DAWA524.198 DAWA609.150c
50,0 074I10_3_1
51,3 DAWA1072.650
53,8 DAWA54.100
55,7 LaSNP_014
56,3 LaSNP_068 LSSR07
59,2 DAWA305.278 IPb-334705 LaIND_016 IPb-332203 IPb-462341 IPb-332892 IPb-449208
59,8 LaSNP_057
60,3 LaIND_223
63,4 DAWA608.170c
64,0 DAWA166.450 DAWA397.400
65,2 MTIC_114
67,7 psat_EST_00178
80,9 051C12_3
86,5 037D21_5D
88,3 DAWA396.550
90,1 DAWA504.280
91,3 DAWA632.550
91,9 lucundus
92,5 DAWA604.335
93,0 DAWA134.265
93,6 DAWA525.185
94,9 IPb-332862 DAWA86.100 DAWA26.250
95,5 051C12_5
98,5 DAWA255.360
99,8 DAWA650.450
100,4 DAWA679.430
127,1 UWA080b
131,0 DAWA619.140
141,3 LaIND_186
143,1 DAWA96.800
145,0 LaIND_105
146,3 DAWA74.050
NLL-08
0,0 LaIND_136 DAWA364.180
1,2 LaIND_086
3,8 DAWA348.400
10,6 IPb-329014 DAWA237.170c
11,3 Lup282
25,0 DAWA15.390 DAWA1075.380
29,7 LSSR01
36,0 LeM2
37,0 Lentus
38,2 DAWA323.150
38,8 DAWA964.275
42,0 DAWA72.160
43,9 IPb-460464 IPb-450055 IPb-448967 IPb-459972 IPb-460104
45,1 LaSSR_034
51,8 DAWA165.480c DAWA155.550c
52,4 DAWA88.325c
55,6 IPb-462410
62,8 LaSSR_002
65,5 DAWA250.170
66,2 dFTb DAWA23.100
68,3 UWA156 DAWA241.295
68,9 084P14_5F2
70,7 142D13_3
73,9 IPb-334581
75,4 DAWA308.198
76,7 Leg208
80,6 DAWA577.150
85,8 IPb-522664 IPb-449158 IPb-460036 IPb-460182
87,5 IPb-329090 IPb-334912 IPb-449449 IPb-334825
94,0 UWA216b
95,9 IPb-522739 IPb-333427
116,2 DAWA275.170
124,0 UWA242c
125,8 IPb-450205 IPb-460016
127,1 UWA215
131,0 DAWA202.180
131,6 LG77
132,3 LG75 DAWA183.175
133,5 DAWA912.325
139,6 psat_EST_00169
146,7 LaSNP_006
NLL-09
0,0 DAWA416.290
0,7 LaIND_142
1,2 LaIND_081
3,8 IPb-332672 IPb-332095
5,5 Lup319
7,7 DAWA265.190
8,9 DAWA274.188
16,0 LaSNP_007 IPb-449572
38,1 Lup233
42,4 mtmt_GEN_00001
44,8 DAWA172.230
46,0 DAWA196.230c
48,5 015P08_3
53,1 Lup241
57,6 CNGC4
58,9 DAWA307.205
60,1 DAWA442.060 80a22-1
62,0 Lup310
62,7 DAWA607.180
75,6 Lup174
83,7 059J08_3
94,5 mtmt_EST_03569
101,7 IPb-449702 IPb-515638 IPb-332084 IPb-449589 IPb-332016 IPb-334142 IPb-449809
102,3 DAWA557.110 Lup088
104,7 LaIND_023
109,0 LaSNP_033
113,0 DAWA195.205
119,3 138H12_5
120,1 061F03_5
124,6 Leg228
131,1 038J06_3

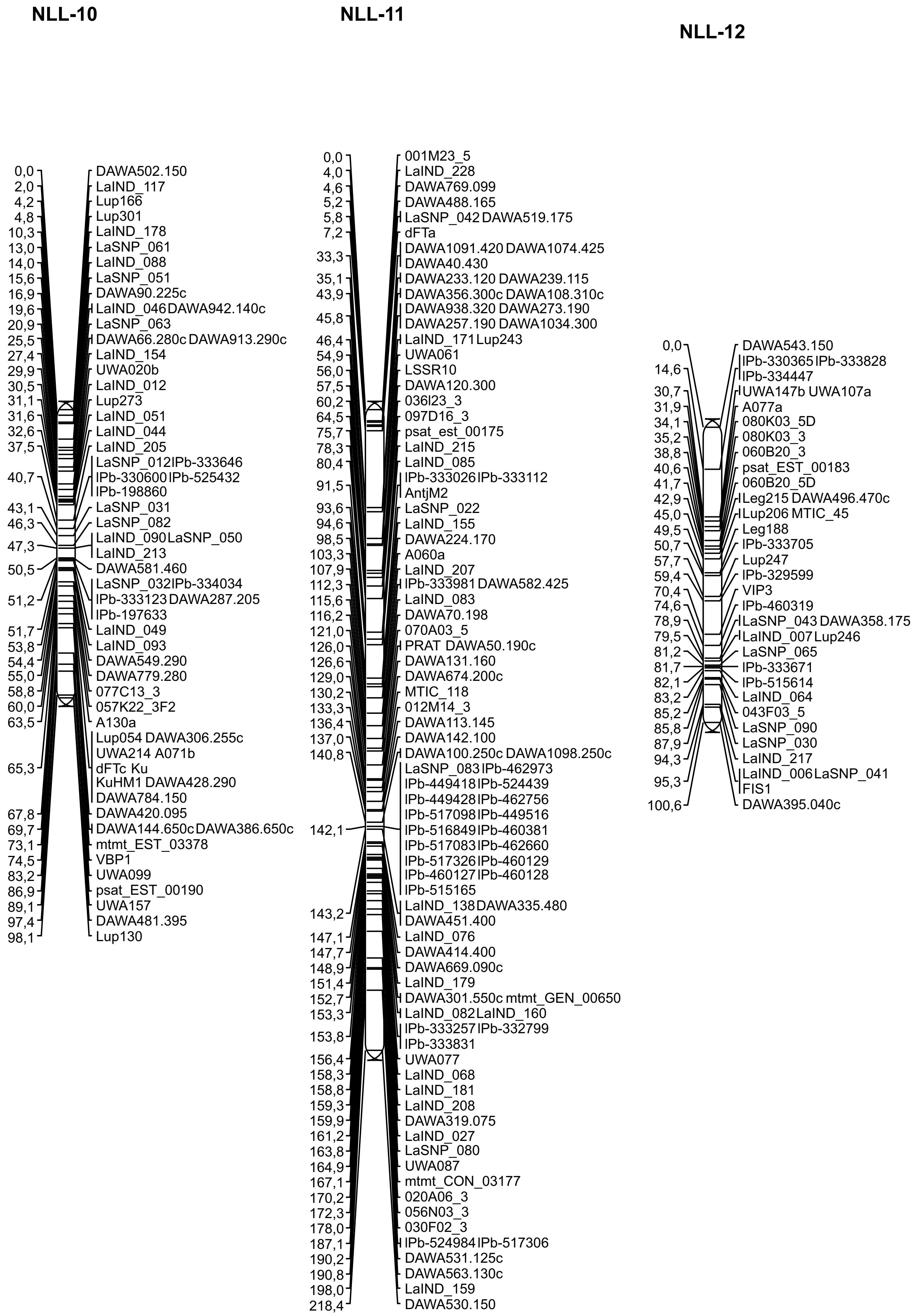
NLL-10
0,0
2,0
4,2
4,8
10,3
13,0
14,0
15,6
16,9
19,6
20,9
25,5
27,4
29,9
30,5
31,1
31,6
32,6
37,5
40,7
43,1
46,3
47,3
50,5
51,2
51,7
53,8
54,4
55,0
58,8
60,0
63,5
65,3
67,8
69,7
73,1
74,5
83,2
86,9
89,1
97,4
98,1
DAWA502.150
LaIND_117
Lup166
Lup301
LaIND_178
LaSNP_061
LaIND_088
LaSNP_051
DAWA90.225c
LaIND_046 DAWA942.140c
LaSNP_063
DAWA66.280c DAWA913.290c
LaIND_154
UWA020b
LaIND_012
Lup273
LaIND_051
LaIND_044
LaIND_205
LaSNP_012 lPb-333646
lPb-330600 lPb-525432
lPb-198860
LaSNP_031
LaSNP_082
LaIND_090 LaSNP_050
LaIND_213
DAWA581.460
LaSNP_032 lPb-334034
lPb-333123 DAWA287.205
lPb-197633
LaIND_049
LaIND_093
DAWA549.290
DAWA779.280
077C13_3
057K22_3F2
A130a
Lup054 DAWA306.255c
UWA214 A071b
dFTc Ku
KuHM1 DAWA428.290
DAWA784.150
DAWA420.095
DAWA144.650c DAWA386.650c
mtmt_EST_03378
VBP1
UWA099
psat_EST_00190
UWA157
DAWA481.395
Lup130
NLL-11
0,0
4,0
4,6
5,2
5,8
7,2
33,3
35,1
43,9
45,8
46,4
54,9
56,0
57,5
60,2
64,5
75,7
78,3
80,4
91,5
93,6
94,6
98,5
103,3
107,9
112,3
115,6
116,2
121,0
126,0
126,6
129,0
130,2
133,3
136,4
137,0
140,8
142,1
143,2
147,1
147,7
148,9
151,4
152,7
153,3
153,8
156,4
158,3
158,8
159,3
159,9
161,2
163,8
164,9
167,1
170,2
172,3
178,0
187,1
190,2
190,8
198,0
218,4
001M23_5
LaIND_228
DAWA769.099
DAWA488.165
LaSNP_042 DAWA519.175
dFTa
DAWA1091.420 DAWA1074.425
DAWA40.430
DAWA233.120 DAWA239.115
DAWA356.300c DAWA108.310c
DAWA938.320 DAWA273.190
DAWA257.190 DAWA1034.300
LaIND_171 Lup243
UWA061
LSSR10
DAWA120.300
036I23_3
097D16_3
psat_est_00175
LaIND_215
LaIND_085
lPb-333026 lPb-333112
AntjM2
LaSNP_022
LaIND_155
DAWA224.170
A060a
LaIND_207
lPb-333981 DAWA582.425
LaIND_083
DAWA70.198
070A03_5
PRAT DAWA50.190c
DAWA131.160
DAWA674.200c
MTIC_118
012M14_3
DAWA113.145
DAWA142.100
DAWA100.250c DAWA1098.250c
LaSNP_083 lPb-462973
lPb-449418 lPb-524439
lPb-449428 lPb-462756
lPb-517098 lPb-449516
lPb-516849 lPb-460381
lPb-517083 lPb-462660
lPb-517326 lPb-460129
lPb-460127 lPb-460128
lPb-515165
LaIND_138 DAWA335.480
DAWA451.400
LaIND_076
DAWA414.400
DAWA669.090c
LaIND_179
DAWA301.550c mtmt_GEN_00650
LaIND_082 LaIND_160
lPb-333257 lPb-332799
lPb-333831
UWA077
LaIND_068
LaIND_181
LaIND_208
DAWA319.075
LaIND_027
LaSNP_080
UWA087
mtmt_CON_03177
020A06_3
056N03_3
030F02_3
lPb-524984 lPb-517306
DAWA531.125c
DAWA563.130c
LaIND_159
DAWA530.150
NLL-12
0,0
14,6
30,7
31,9
34,1
35,2
38,8
40,6
41,7
42,9
45,0
49,5
50,7
57,7
59,4
70,4
74,6
78,9
79,5
81,2
81,7
82,1
83,2
85,2
85,8
87,9
94,3
95,3
100,6
DAWA543.150
lPb-330365 lPb-333828
lPb-334447
UWA147b UWA107a
A077a
080K03_5D
080K03_3
060B20_3
psat_EST_00183
060B20_5D
Leg215 DAWA496.470c
Lup206 MTIC_45
Leg188
lPb-333705
Lup247
lPb-329599
VIP3
lPb-460319
LaSNP_043 DAWA358.175
LaIND_007 Lup246
LaSNP_065
lPb-333671
lPb-515614
LaIND_064
043F03_5
LaSNP_090
LaSNP_030
LaIND_217
LaIND_006 LaSNP_041
FIS1
DAWA395.040c

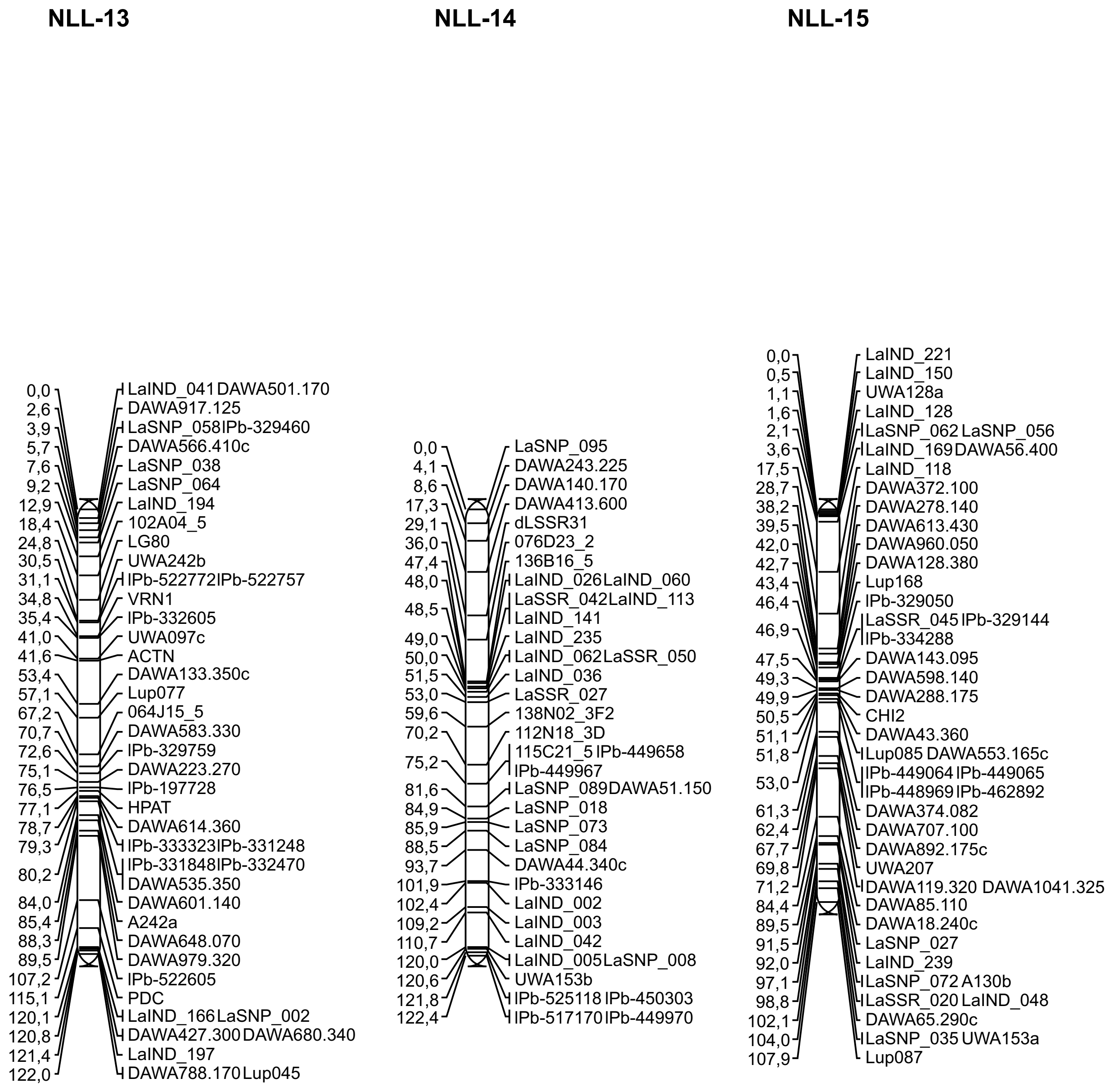
NLL-13
0,0 LaIND_041 DAWA501.170
2,6 DAWA917.125
3,9 LaSNP_058 IPb-329460
5,7 DAWA566.410c
7,6 LaSNP_038
9,2 LaSNP_064
12,9 LaIND_194
18,4 102A04_5
24,8 LG80
30,5 UWA242b
31,1 IPb-522772 IPb-522757
34,8 VRN1
35,4 IPb-332605
41,0 UWA097c
41,6 ACTN
53,4 DAWA133.350c
57,1 Lup077
67,2 064J15_5
70,7 DAWA583.330
72,6 IPb-329759
75,1 DAWA223.270
76,5 IPb-197728
77,1 HPAT
78,7 DAWA614.360
79,3 IPb-333323 IPb-331248
80,2 IPb-331848 IPb-332470 DAWA535.350
84,0 DAWA601.140
85,4 A242a
88,3 DAWA648.070
89,5 DAWA979.320
107,2 IPb-522605
115,1 PDC
120,1 LaIND_166 LaSNP_002
120,8 DAWA427.300 DAWA680.340
121,4 LaIND_197
122,0 DAWA788.170 Lup045
NLL-14
0,0 LaSNP_095
4,1 DAWA243.225
8,6 DAWA140.170
17,3 DAWA413.600
29,1 dLSSR31
36,0 076D23_2
47,4 136B16_5
48,0 LaIND_026 LaIND_060
48,5 LaSSR_042 LaIND_113 LaIND_141
49,0 LaIND_235
50,0 LaIND_062 LaSSR_050
51,5 LaIND_036
53,0 LaSSR_027
59,6 138N02_3F2
70,2 112N18_3D
75,2 115C21_5 IPb-449658 IPb-449967
81,6 LaSNP_089 DAWA51.150
84,9 LaSNP_018
85,9 LaSNP_073
88,5 LaSNP_084
93,7 DAWA44.340c
101,9 IPb-333146
102,4 LaIND_002
109,2 LaIND_003
110,7 LaIND_042
120,0 LaIND_005 LaSNP_008
120,6 UWA153b
121,8 IPb-525118 IPb-450303
122,4 IPb-517170 IPb-449970
NLL-15
0,0 LaIND_221
0,5 LaIND_150
1,1 UWA128a
1,6 LaIND_128
2,1 LaSNP_062 LaSNP_056
3,6 LaIND_169 DAWA56.400
17,5 LaIND_118
28,7 DAWA372.100
38,2 DAWA278.140
39,5 DAWA613.430
42,0 DAWA960.050
42,7 DAWA128.380
43,4 Lup168
46,4 IPb-329050
46,9 LaSSR_045 IPb-329144 IPb-334288
47,5 DAWA143.095
49,3 DAWA598.140
49,9 DAWA288.175
50,5 CHI2
51,1 DAWA43.360
51,8 Lup085 DAWA553.165c
53,0 IPb-449064 IPb-449065 IPb-448969 IPb-462892
61,3 DAWA374.082
62,4 DAWA707.100
67,7 DAWA892.175c
69,8 UWA207
71,2 DAWA119.320 DAWA1041.325
84,4 DAWA85.110
89,5 DAWA18.240c
91,5 LaSNP_027
92,0 LaIND_239
97,1 LaSNP_072 A130b
98,8 LaSSR_020 LaIND_048
102,1 DAWA65.290c
104,0 LaSNP_035 UWA153a
107,9 Lup087

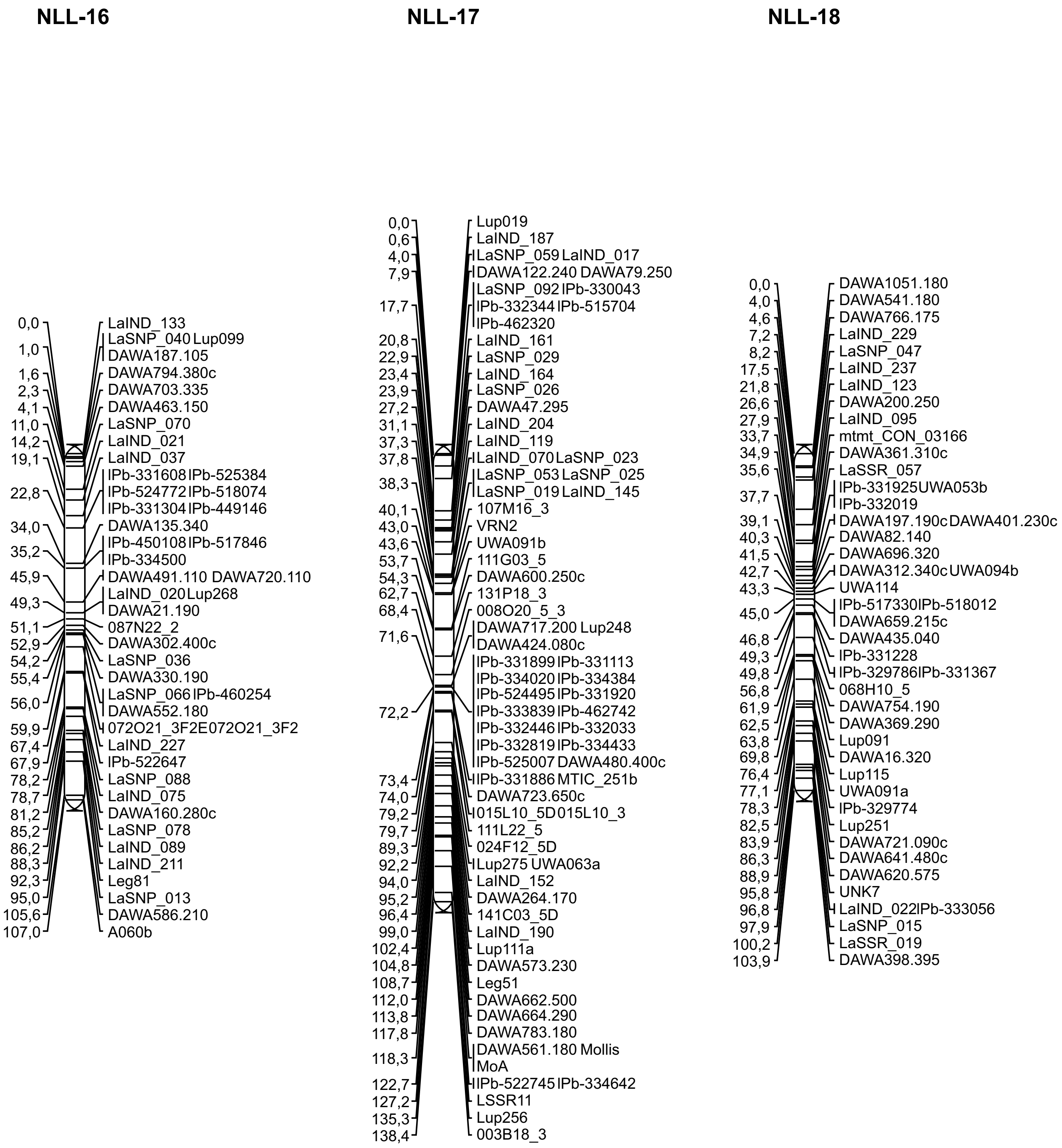
NLL-16
0,0
1,0
1,6
2,3
4,1
11,0
14,2
19,1
22,8
34,0
35,2
45,9
49,3
51,1
52,9
54,2
55,4
56,0
59,9
67,4
67,9
78,2
78,7
81,2
85,2
86,2
88,3
92,3
95,0
105,6
107,0
LaIND_133
LaSNP_040 Lup099
DAWA187.105
DAWA794.380c
DAWA703.335
DAWA463.150
LaSNP_070
LaIND_021
LaIND_037
IPb-331608 IPb-525384
IPb-524772 IPb-518074
IPb-331304 IPb-449146
DAWA135.340
IPb-450108 IPb-517846
IPb-334500
DAWA491.110 DAWA720.110
LaIND_020 Lup268
DAWA21.190
087N22_2
DAWA302.400c
LaSNP_036
DAWA330.190
LaSNP_066 IPb-460254
DAWA552.180
072O21_3F2E072O21_3F2
LaIND_227
IPb-522647
LaSNP_088
LaIND_075
DAWA160.280c
LaSNP_078
LaIND_089
LaIND_211
Leg81
LaSNP_013
DAWA586.210
A060b
NLL-17
0,0
0,6
4,0
7,9
17,7
20,8
22,9
23,4
23,9
27,2
31,1
37,3
37,8
38,3
40,1
43,0
43,6
53,7
54,3
62,7
68,4
71,6
72,2
73,4
74,0
79,2
79,7
89,3
92,2
94,0
95,2
96,4
99,0
102,4
104,8
108,7
112,0
113,8
117,8
118,3
122,7
127,2
135,3
138,4
Lup019
LaIND_187
LaSNP_059 LaIND_017
DAWA122.240 DAWA79.250
LaSNP_092 IPb-330043
IPb-332344 IPb-515704
IPb-462320
LaIND_161
LaSNP_029
LaIND_164
LaSNP_026
DAWA47.295
LaIND_204
LaIND_119
LaIND_070 LaSNP_023
LaSNP_053 LaSNP_025
LaSNP_019 LaIND_145
107M16_3
VRN2
UWA091b
111G03_5
DAWA600.250c
131P18_3
008O20_5_3
DAWA717.200 Lup248
DAWA424.080c
IPb-331899 IPb-331113
IPb-334020 IPb-334384
IPb-524495 IPb-331920
IPb-333839 IPb-462742
IPb-332446 IPb-332033
IPb-332819 IPb-334433
IPb-525007 DAWA480.400c
IPb-331886 MTIC_251b
DAWA723.650c
015L10_5D 015L10_3
111L22_5
024F12_5D
Lup275 UWA063a
LaIND_152
DAWA264.170
141C03_5D
LaIND_190
Lup111a
DAWA573.230
Leg51
DAWA662.500
DAWA664.290
DAWA783.180
DAWA561.180 Mollis
MoA
IPb-522745 IPb-334642
LSSR11
Lup256
003B18_3
NLL-18
0,0
4,0
4,6
7,2
8,2
17,5
21,8
26,6
27,9
33,7
34,9
35,6
37,7
39,1
40,3
41,5
42,7
43,3
45,0
46,8
49,3
49,8
56,8
61,9
62,5
63,8
69,8
76,4
77,1
78,3
82,5
83,9
86,3
88,9
95,8
96,8
97,9
100,2
103,9
DAWA1051.180
DAWA541.180
DAWA766.175
LaIND_229
LaSNP_047
LaIND_237
LaIND_123
DAWA200.250
LaIND_095
mtmt_CON_03166
DAWA361.310c
LaSSR_057
IPb-331925UWA053b
IPb-332019
DAWA197.190c DAWA401.230c
DAWA82.140
DAWA696.320
DAWA312.340c UWA094b
UWA114
IPb-517330IPb-518012
DAWA659.215c
DAWA435.040
IPb-331228
IPb-329786IPb-331367
068H10_5
DAWA754.190
DAWA369.290
Lup091
DAWA16.320
Lup115
UWA091a
IPb-329774
Lup251
DAWA721.090c
DAWA641.480c
DAWA620.575
UNK7
LaIND_022IPb-333056
LaSNP_015
LaSSR_019
DAWA398.395

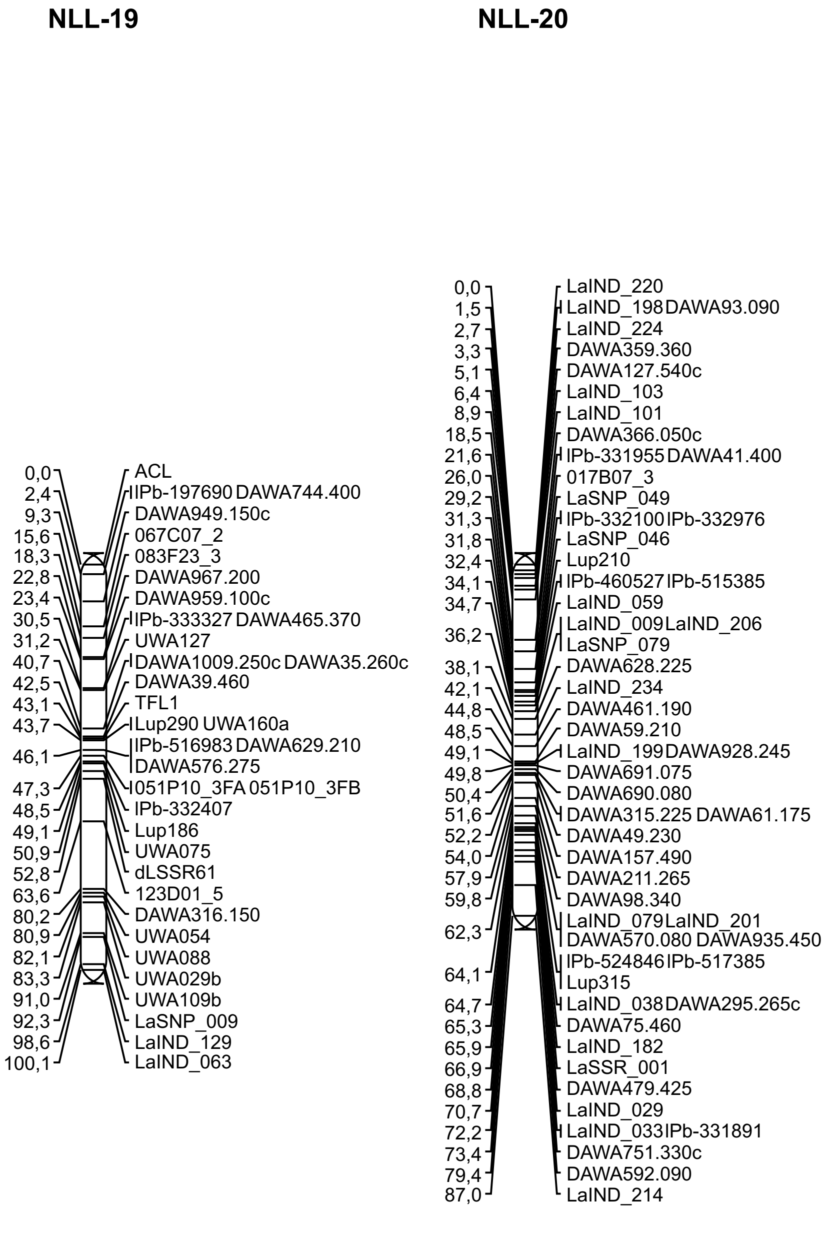
NLL-19
NLL-20
0,0 ACL
2,4 IPb-197690 DAWA744.400
9,3 DAWA949.150c
15,6 067C07_2
18,3 083F23_3
22,8 DAWA967.200
23,4 DAWA959.100c
30,5 IPb-333327 DAWA465.370
31,2 UWA127
40,7 DAWA1009.250c DAWA35.260c
42,5 DAWA39.460
43,1 TFL1
43,7 Lup290 UWA160a
46,1 IPb-516983 DAWA629.210
DAWA576.275
47,3 051P10_3FA 051P10_3FB
48,5 IPb-332407
49,1 Lup186
50,9 UWA075
52,8 dLSSR61
63,6 123D01_5
80,2 DAWA316.150
80,9 UWA054
82,1 UWA088
83,3 UWA029b
91,0 UWA109b
92,3 LaSNP_009
98,6 LaIND_129
100,1 LaIND_063
0,0 LaIND_220
1,5 LaIND_198 DAWA93.090
2,7 LaIND_224
3,3 DAWA359.360
5,1 DAWA127.540c
6,4 LaIND_103
8,9 LaIND_101
18,5 DAWA366.050c
21,6 IPb-331955 DAWA41.400
26,0 017B07_3
29,2 LaSNP_049
31,3 IPb-332100 IPb-332976
31,8 LaSNP_046
32,4 Lup210
34,1 IPb-460527 IPb-515385
34,7 LaIND_059
36,2 LaIND_009 LaIND_206
LaSNP_079
38,1 DAWA628.225
42,1 LaIND_234
44,8 DAWA461.190
48,5 DAWA59.210
49,1 LaIND_199 DAWA928.245
49,8 DAWA691.075
50,4 DAWA690.080
51,6 DAWA315.225 DAWA61.175
52,2 DAWA49.230
54,0 DAWA157.490
57,9 DAWA211.265
59,8 DAWA98.340
62,3 LaIND_079 LaIND_201
DAWA570.080 DAWA935.450
64,1 IPb-524846 IPb-517385
Lup315
64,7 LaIND_038 DAWA295.265c
65,3 DAWA75.460
65,9 LaIND_182
66,9 LaSSR_001
68,8 DAWA479.425
70,7 LaIND_029
72,2 LaIND_033 IPb-331891
73,4 DAWA751.330c
79,4 DAWA592.090
87,0 LaIND_214
