# Supplementary figures and images for: Integration of Lupinus angustifolius L. (narrow-leafed lupin) genome maps and comparative mapping within legumes
Source: Chromosome Res. 2016 May 11;24:355–78. doi: 10.1007/s10577-016-9526-8 (PMC4969343; doi:10.1007/s10577-016-9526-8)

A 14 BACs

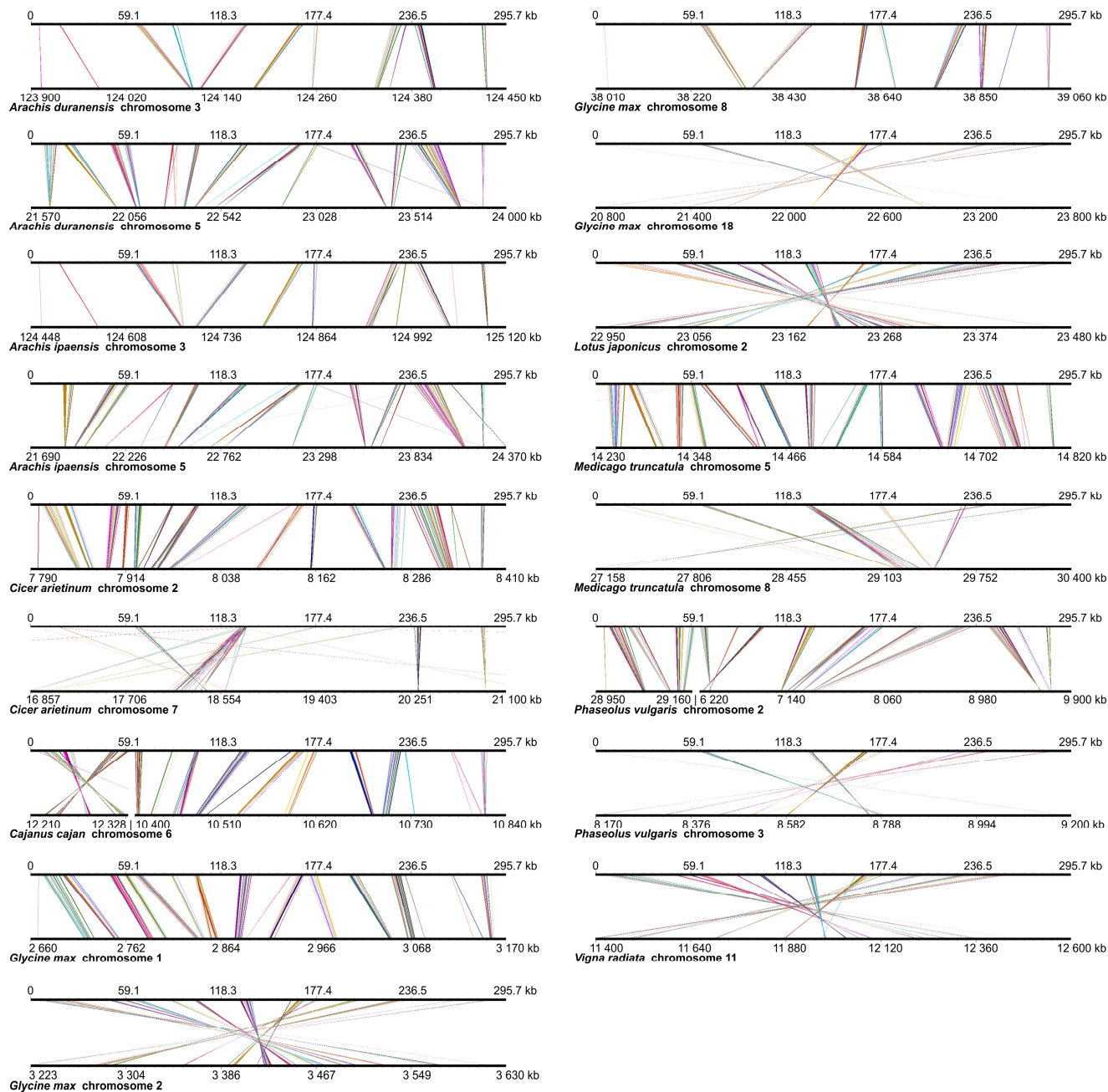

B 059F07

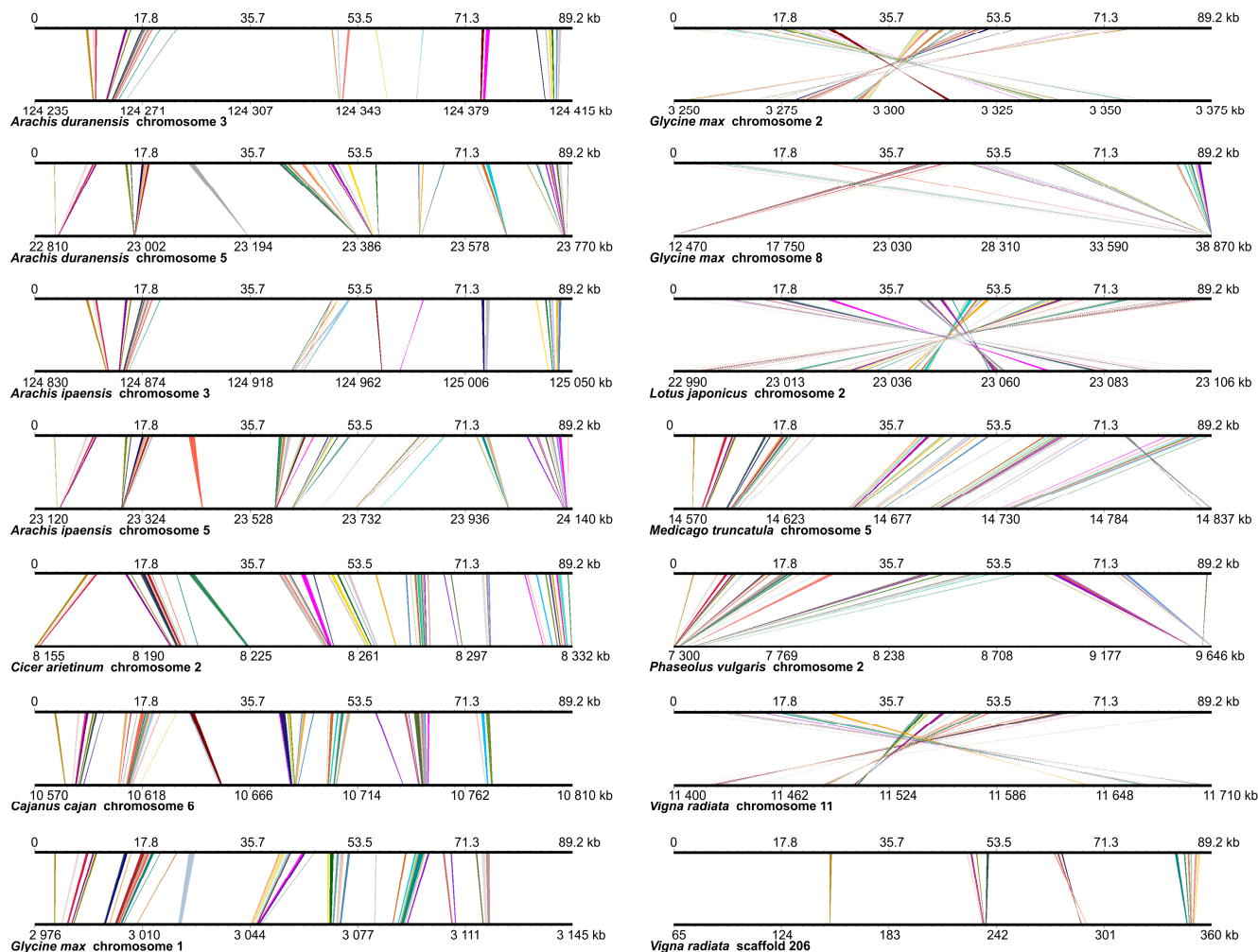

C 068H10

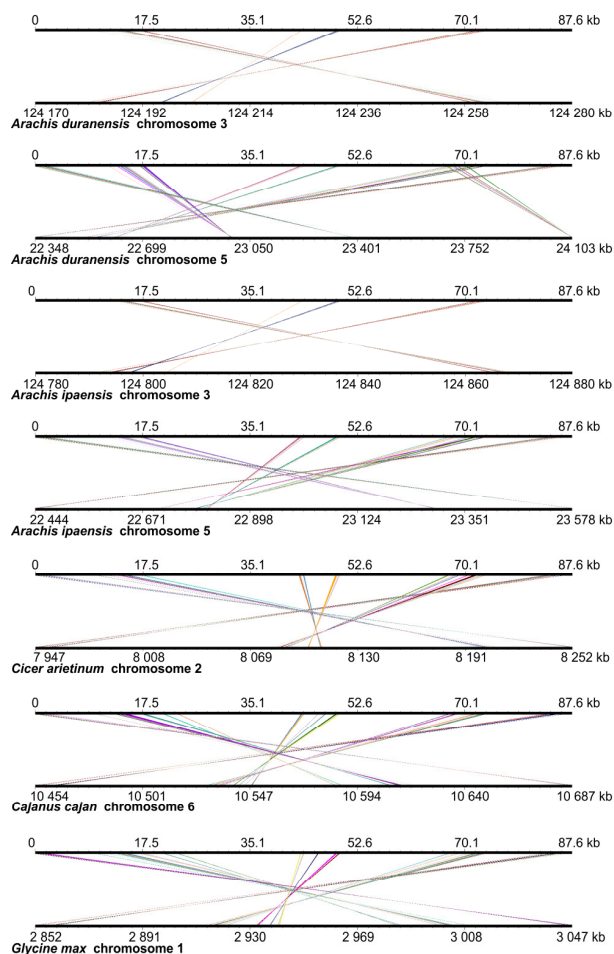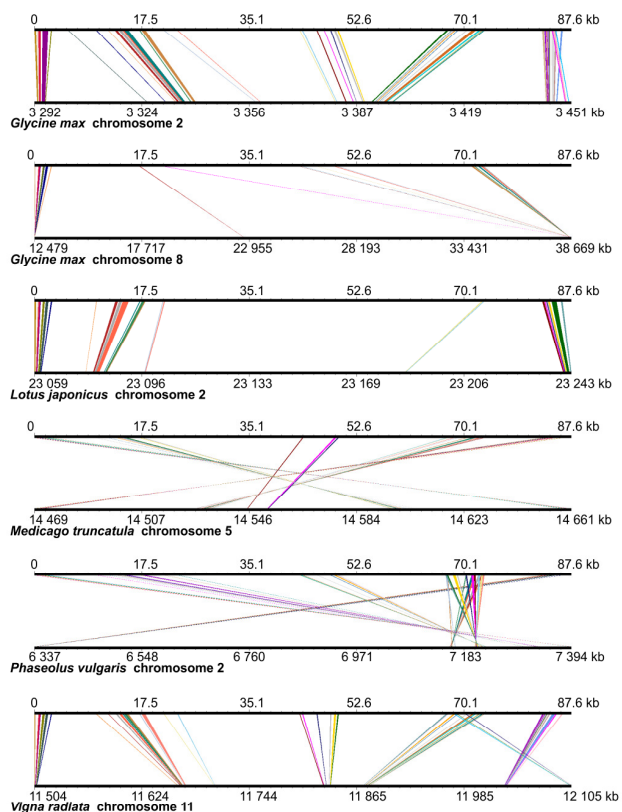

D 087F06

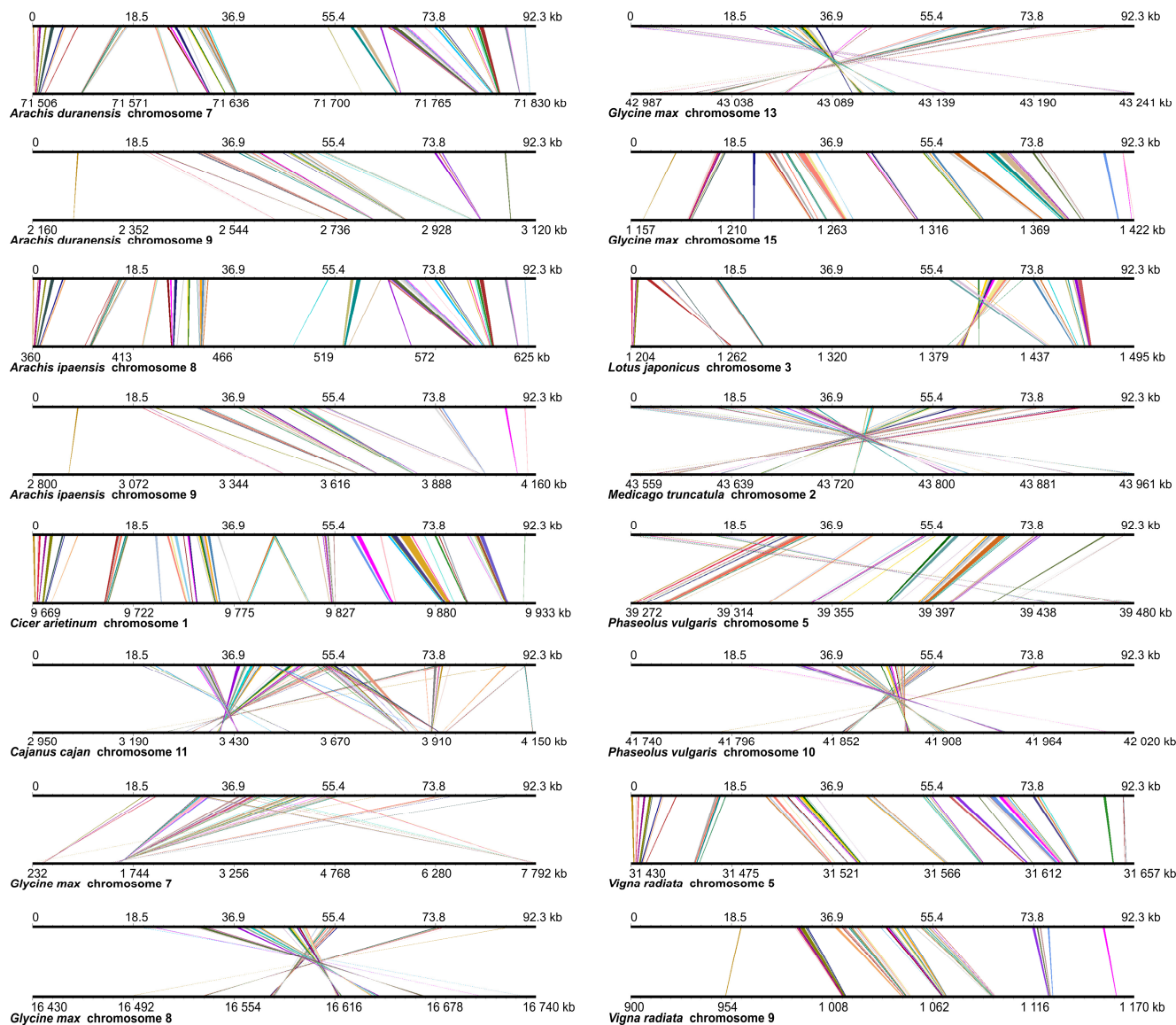

E 102A04

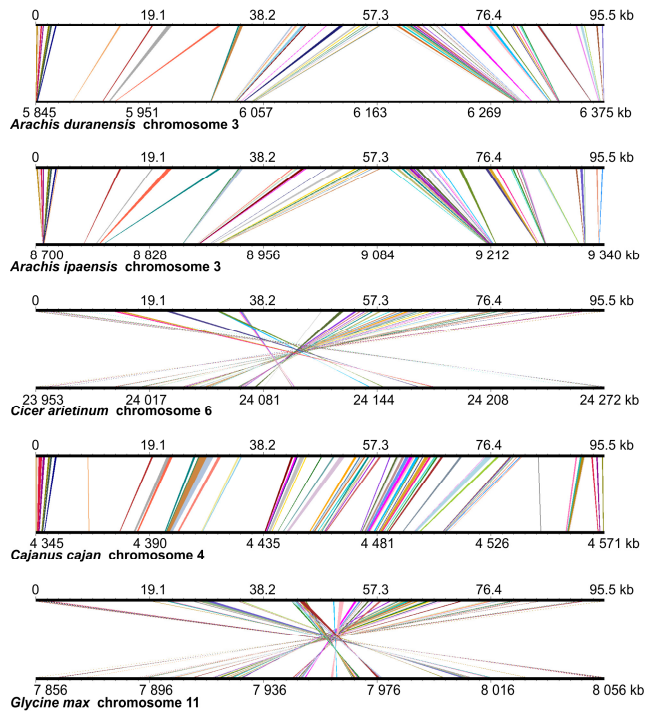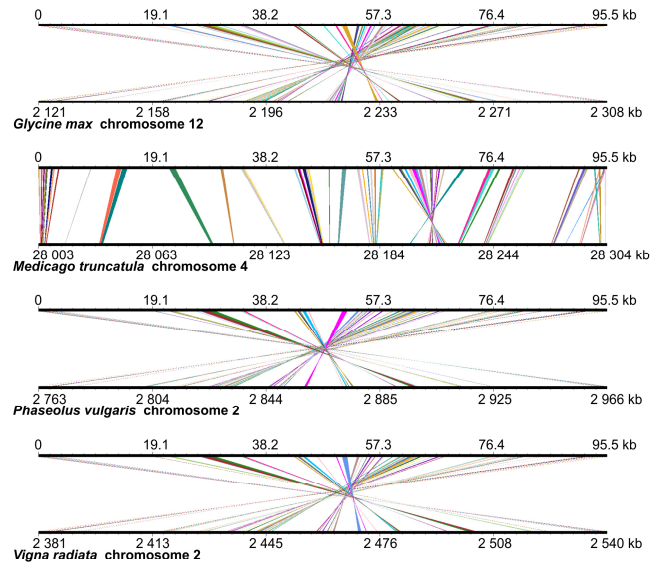

F 127N17

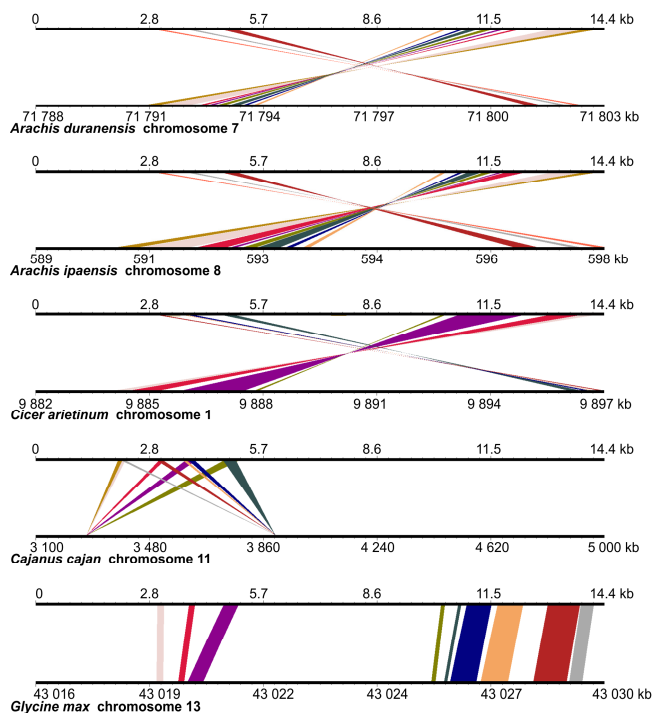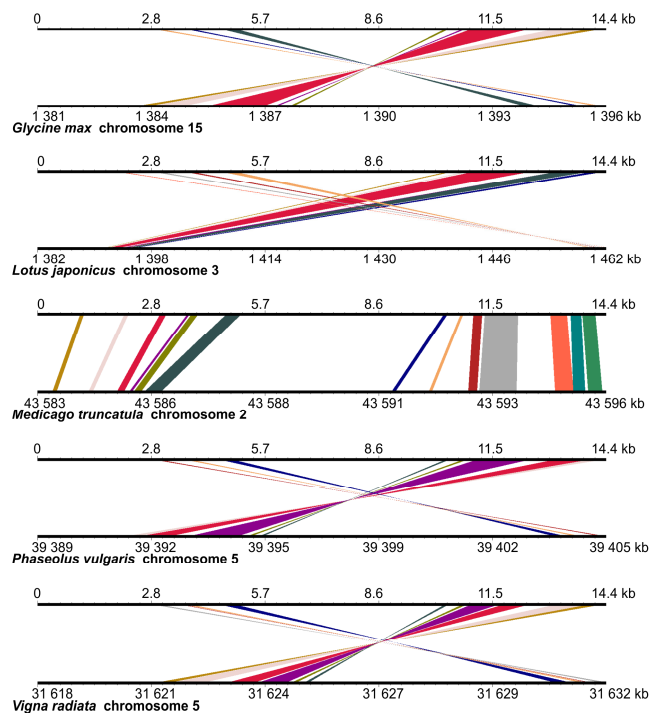

G 138H12

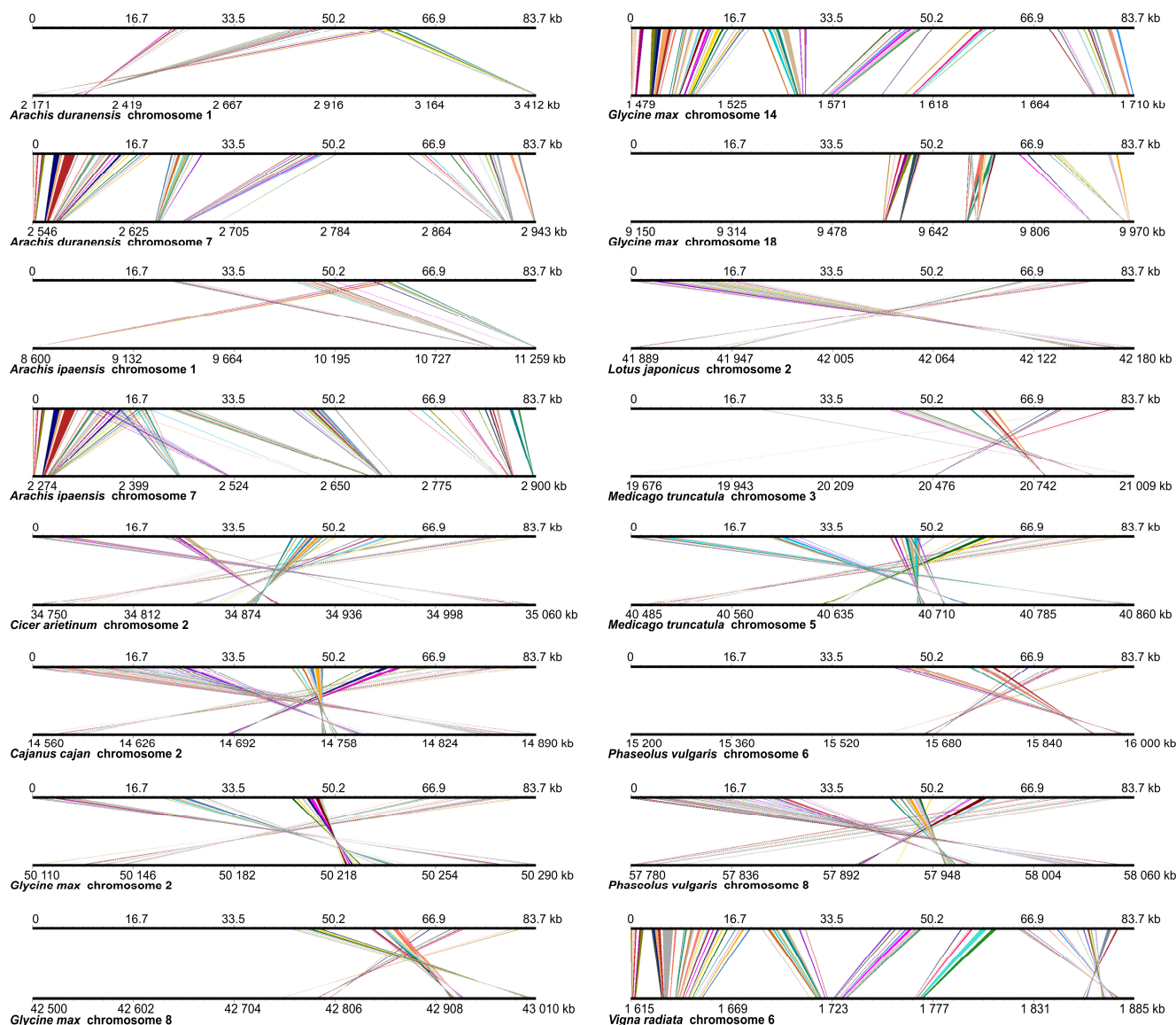

Supplement: Supplementary file 14 — Graphical representation of the most conserved syntenic blocks identified between a 131P18, b 059F07, c 068H10, d 087F06, e 102A04, f 127N17, and g 138H12 for nine legume species. (PDF 15,164 kb) [file 10577_2016_9526_MOESM14_ESM.pdf]
